# Mechanistic insights into Strontium-90 superficial radiotherapy in keloids: senescence-associated changes and ECM remodeling across cell-matrix scales

**DOI:** 10.3389/fphar.2026.1832367

**Published:** 2026-08-19

**Authors:** Fushi Han, Shengnan Ren, Xiaoran Mei, Linlin Li, Wenyu Zhao, Fang Feng

**Affiliations:** Department of Nuclear Medicine, Shanghai Fourth People’s Hospital Affiliated to Tongji University, Shanghai, China

**Keywords:** cellular senescence, keloid, LOX/LOXL2, pharmacological mechanism, strontium-90 radiotherapy

## Abstract

**Background:**

The limited efficacy of current keloid therapies necessitates a deeper understanding of treatment mechanisms. This study investigates the therapeutic potential and underlying pharmacological actions of strontium-90 (Sr-90) superficial radiotherapy, evaluating a dual-component antifibrotic working model.

**Methods:**

The effects of Sr-90 irradiation were studied in primary keloid fibroblasts and a rabbit ear scar model. We assessed cell proliferation, migration, senescence (SA-β-gal), and DNA damage. Molecular profiling (Western blot, RT-qPCR, enzymatic assays) evaluated the p53-p21-DNA damage response axis and lysyl oxidase (LOX/LOXL2) pathway. Transcriptomic analysis (RNA-seq) with GO/KEGG enrichment was performed. Collagen ultrastructure and crosslinking-related indices were analyzed, alongside tissue remodeling evaluated by multimodal imaging optical coherence tomography and biomechanics (nanoindentation). Functional validation used Pifithrin-α and β-aminopropionitrile (BAPN).

**Results:**

Sr-90 irradiation was accompanied by activation of the p53-p21-DDR pathway, increased senescence-associated features, and reduced fibroblast proliferation and migration. Concurrently, it suppressed LOX/LOXL2 expression and activity, accompanied by changes in collagen crosslinking-related indices and improved tissue elasticity. Transcriptomics revealed downregulation of genes in extracellular matrix (ECM) organization, focal adhesion, and PI3K-Akt signaling. These changes were accompanied by reduced collagen deposition, disassembly of the collagen network, decreased fibrotic thickness, and restoration of tissue mechanical properties. P53 pathway inhibition attenuated these effects, whereas LOX inhibition further enhanced the matrix-remodeling response. Animal findings were generally consistent with the *in vitro* observations.

**Conclusion:**

Sr-90 irradiation was associated with fibroblast senescence and accompanying p53-p21-DDR activation, together with reduced LOX/LOXL2 expression and activity, which may contribute to attenuation of collagen crosslinking-related matrix stabilization. These parallel cellular and matrix-associated changes may jointly promote scar remodeling by affecting both fibroblast behavior and ECM architecture. These findings provide preliminary mechanistic support for further investigation of Sr-90 superficial radiotherapy and matrix-targeted combination strategies in keloid fibrosis.

## Graphical Abstract

**Figure:**
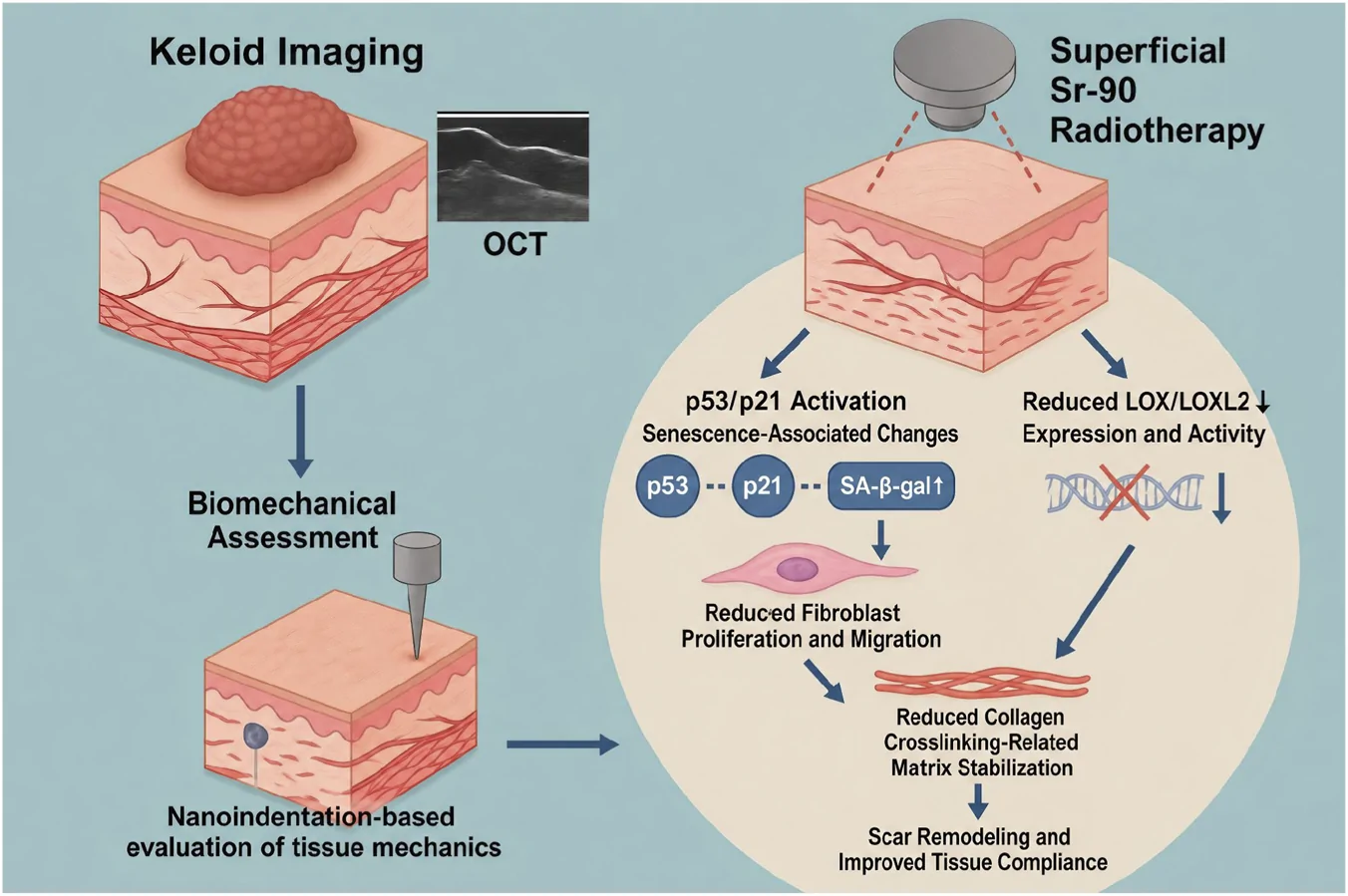
Proposed working model of parallel senescence-associated and collagen crosslinking-related ECM changes following Sr-90 superficial radiotherapy in keloid tissue.

## Highlights

- Sr-90 radiotherapy was associated with senescence-related changes and attenuation of collagen crosslinking-related matrix remodeling.
- A cross-scale evaluation framework integrates OCT, nanoindentation, SEM, and molecular profiling.
- Sr-90 irradiation was accompanied by p53-p21-DDR activation, senescence-associated changes, and reduced fibroblast proliferation and migration.
- Radiotherapy downregulated LOX/LOXL2 expression and enzymatic activity, together with changes in collagen crosslinking-related indices and tissue mechanical properties.
- Combination treatment with BAPN produced a stronger combined effect, with broadly consistent observations in both *in vitro* and *in vivo* models.

## Introduction

Keloids are pathological scars that arise from aberrant healing following cutaneous injury, characterized clinically by persistent growth beyond the original wound margins and a high propensity for recurrence, often accompanied by pruritus, pain, and marked cosmetic disfigurement, all of which significantly compromise quality of life (Kim and Kim, 2024; Delaleu et al., 2023). Unlike mature scars, keloids do not simply reflect excessive collagen deposition; rather, they represent a complex fibrotic disorder driven by aberrant fibroblast activation, abnormal extracellular matrix (ECM) accumulation, and altered mechanical microenvironments (Kohlhauser et al., 2024; Kim and Kim, 2024; Kim et al., 2024). Previous studies have demonstrated that keloid fibroblasts exhibit excessive proliferation, enhanced migratory capacity, resistance to apoptosis, and heightened secretory activity, and that their persistent crosstalk with ECM and inflammatory signals sustains a profibrotic state (Jiao et al., 2023; Li et al., 2023; Chao et al., 2023). Accordingly, a mechanistic investigation centered on dysregulated fibroblasts and their matrix microenvironment is essential for understanding keloid pathogenesis and identifying effective therapeutic targets (Kohlhauser et al., 2024; Fang et al., 2025).

Current treatment options for keloids include surgical excision, intralesional corticosteroid injection, laser therapy, pressure therapy, and radiotherapy; however, clinical management remains limited by high recurrence rates, inconsistent efficacy, and substantial interindividual variability (Liu et al., 2023; Frech et al., 2023; Fang et al., 2025). Sr-90 radiotherapy, as an adjunctive treatment, has been applied in clinical practice, particularly for controlling local recurrence following combined therapies (Deng et al., 2021; Nien et al., 2025). Nevertheless, existing studies have largely focused on its effects on local recurrence control or cell proliferation inhibition, and systematic mechanistic evidence is still lacking regarding whether Sr-90 superficial radiotherapy can concurrently modulate fibroblast states and ECM remodeling to elicit a stronger antifibrotic effect (Deng et al., 2021; Nien et al., 2025; Ji et al., 2015). Whether the therapeutic value of Sr-90 extends beyond suppressing fibroblast proliferation and recurrence to active remodeling of scar tissue remains unclear (Ji et al., 2015; Deng et al., 2021; Kim and Kim, 2024).

Mechanistically, ionizing radiation can trigger the DNA damage response (DDR) and activate canonical signaling pathways, such as p53/p21, resulting in cell-cycle arrest, proliferation inhibition, and ultimately induction of cellular senescence (Guan et al., 2025; Saini and Gurung, 2025). Because radiation-induced senescence is classically linked to persistent DDR activation and p53/p21-dependent growth arrest, and because Pifithrin-α has been widely used to inhibit p53 transcriptional activity, the p53/p21 axis was selected as the principal pathway for evaluating Sr-90-induced fibroblast senescence in this study (Zhu et al., 2020). In recent years, cellular senescence has emerged as a potentially protective mechanism that constrains the persistent expansion of aberrantly activated fibroblasts in multiple fibrotic disorders (O'Reilly et al., 2024; Jipu et al., 2025). In the context of keloids, inducing pathologically activated fibroblasts into a senescent state may attenuate their sustained capacity for collagen synthesis and matrix remodeling at the source (Ji et al., 2015; Kong et al., 2025).

The refractoriness of scar tissue is not solely attributable to increased collagen deposition, but also to enhanced collagen crosslinking, dense fiber bundle packing, and elevated tissue stiffness (Lloyd and He, 2024; Feng et al., 2022; He et al., 2024). Collagen crosslinking mediated by LOX and LOXL2 constitutes a major molecular basis for matrix stabilization and mechanical aberrancy, while matrix stiffening in turn further promotes fibroblast activation, establishing a vicious cycle of reciprocal reinforcement between cellular dysregulation and matrix hardening (Poe et al., 2023; Kim et al., 2021; He et al., 2024). Therefore, coordinated intervention targeting both fibroblast behavior and matrix architecture may be critical for enhancing antifibrotic efficacy (Lloyd and He, 2024; Feng et al., 2022). On this basis, we hypothesized that the antifibrotic effects of Sr-90 superficial radiotherapy may be represented by a dual-component working model involving p53/p21-associated senescence-related changes and reduced LOX/LOXL2-associated matrix stabilization.

Traditional mechanistic studies and therapeutic evaluations have relied predominantly on histopathology, proliferation assays, or single molecular markers, which are insufficient to capture the continuous and interconnected changes from molecular regulation and cellular state to ECM architecture and tissue mechanics (Su et al., 2025; Zhao et al., 2025). Keloids involve remodeling across multiple biological scales, including intracellular stress signaling, ECM reorganization, and tissue-level mechanical abnormalities. This complexity requires an evaluation framework that covers molecular, cellular, matrix, and tissue-level functional changes (Cheng et al., 2024; Su et al., 2025). The integration of multimodal imaging, biomechanical testing, and transcriptomic profiling provides a novel strategy for systematically delineating post-radiotherapy structural and functional remodeling, while also enabling the identification of key molecular networks and tissue-level effects (Meng et al., 2023).

This study examined whether Sr-90 superficial radiotherapy is associated with parallel changes in fibroblast senescence and collagen crosslinking-related matrix remodeling. Using primary keloid fibroblasts and a rabbit ear scar model, we systematically evaluated the effects of Sr-90 on fibroblast viability, proliferation, migration, senescence phenotypes, DDR, and collagen secretion, with particular emphasis on the regulatory roles of the p53/p21 pathway and the LOX/LOXL2 axis. By integrating transcriptomics, multimodal imaging, and biomechanical analysis, we established a cross-scale evaluation framework to delineate how Sr-90 superficial radiotherapy reshapes fibroblast behavior, crosslinking-related matrix indices, and the tissue mechanical microenvironment. Key molecular events and the effects of combination treatment with an LOX inhibitor were further evaluated through pathway blockade and combined intervention experiments. These findings broaden understanding of the antifibrotic effects of Sr-90 superficial radiotherapy from the perspective of cell-matrix regulation. They also provide preliminary mechanistic information that may inform future dose-response studies and the design of combination antifibrotic strategies in keloids.

## Materials and methods

### Isolation and culture of primary keloid fibroblasts

Primary keloid fibroblasts were isolated, cultured, and subjected to superficial Sr-90 irradiation under aseptic conditions. Fresh keloid samples were collected during surgery after obtaining written informed consent from patients and were immediately transported to the laboratory in sterile saline. In a laminar-flow cabinet, the tissues were cut into ∼1 mm^3^ pieces with sterile surgical scissors, followed by digestion in 0.1% type II collagenase solution (Gibco, Cat#17101015, United States) at 37 °C for 6–8 h with gentle shaking. The digested suspension was filtered through a 70 μm cell strainer (Corning, Cat#352350, United States) to remove undigested tissue fragments. The filtrate was then centrifuged at 300 *g* for 5 min, and the resulting cell pellet was resuspended in high-glucose DMEM (Gibco, Cat#11965092, United States) containing 10% FBS (Gibco, Cat#10099141C, United States) and 1% penicillin-streptomycin (Gibco, Cat#15140122, United States). Cells were plated in T25 flasks and cultured at 37 °C in a humidified incubator with 5% CO_2_. When the cells reached 80%–90% confluence, they were subcultured using 0.25% trypsin-EDTA (Gibco, Cat#25200072, United States). Fibroblasts from passages 3–5 were used for subsequent experiments.

To evaluate the biological effects of superficial β-irradiation on scar fibroblasts, a single dose of superficial irradiation was delivered using an Sr-90 applicator (GE Healthcare; customized β-source module; dose rate determined experimentally using a radiation dosimeter) at 2 Gy. The 2 Gy dose was selected as an exploratory low-dose exposure for mechanistic evaluation rather than as a clinical fractionation regimen. This dose was chosen to minimize extensive acute cytotoxicity while allowing assessment of DDR activation, senescence-associated phenotypic changes, and ECM remodeling-related molecular responses under a controlled *in vitro* setting. The delivered dose was verified using a radiation dosimeter before the formal experiment. During irradiation, cultured cells were evenly seeded onto presterilized glass coverslips (Thermo Fisher, Cat#12–545–80, United States) to form a uniform adherent monolayer and were then exposed to the Sr-90 radiation source for homogeneous irradiation. Control cells were cultured under identical conditions but received no irradiation.

### Immunofluorescence (IF) staining

Keloid fibroblasts grown on coverslips were fixed with 4% paraformaldehyde (Solarbio, Cat#P1110, China) for 15 min at room temperature, followed by permeabilization using 0.3% Triton X-100 (Sigma-Aldrich, Cat#T8787, United States) for 10 min. After three washes with PBS (Gibco, Cat#10010023, United States), cells were blocked for 1 h at room temperature in PBS supplemented with 5% FBS (Gibco, Cat#10099141C, United States) to reduce nonspecific binding. Subsequently, samples were incubated overnight at 4 °C in a humidified chamber with primary antibodies, including anti-γ-H2AX (phospho-histone H2AX [Ser139], Cell Signaling Technology, Cat#9718, United States; 1:500), mouse anti-α-SMA monoclonal antibody (Abcam, Cat#ab7817, United Kingdom; 1:500), rabbit anti-IL-6 antibody (Abcam, Cat#ab233706, United Kingdom; 1:200), rabbit anti-IL-8 antibody (Abcam, Cat#ab7747, United Kingdom; 1:200), rabbit anti-LOX antibody (Abcam, Cat#ab31238, United Kingdom; 1:200), and mouse anti-LOXL2 antibody (R&D Systems, Cat#MAB2639, United States; 1:200). On the following day, samples were rinsed three times with PBS for 5 min each and then incubated for 1 h in the dark with either Alexa Fluor 594-conjugated goat anti-rabbit secondary antibody (Thermo Fisher, Cat#A-11012, United States; 1:1,000) or Alexa Fluor 594-conjugated goat anti-mouse IgG secondary antibody (Thermo Fisher, Cat#A-11005, United States; 1:1,000). After an additional three PBS washes, nuclei were counterstained and mounted using an antifade mounting medium containing DAPI (Vector Laboratories, Cat#H-1200, United States). Images were captured using a confocal laser scanning microscope (Leica TCS SP8, Germany) under identical acquisition settings for all experimental groups.

### SA-β-gal staining

Cellular senescence was evaluated using a commercial Senescence β-Galactosidase Staining Kit (Cell Signaling Technology, Cat#9860, United States). Keloid fibroblasts at passages 3–5 were seeded onto coverslips in 24-well plates and grown until reaching approximately 70%–80% confluence. The medium was then discarded, and cells were fixed with the provided fixative for 15 min, followed by two washes at room temperature. Subsequently, cells were incubated with freshly prepared staining solution containing X-gal in a buffer at pH 6.0, ensuring complete coverage of the cell layer. The incubation was carried out at 37 °C in a humidified, CO_2_-free environment protected from light for 12–16 h. After staining, cells were gently washed with PBS and examined under an inverted light microscope (Olympus IX73, Japan), followed by image acquisition. Senescent cells were quantified by randomly selecting multiple fields of view and calculating the percentage of SA-β-gal-positive cells.

### CCK-8 assay for cell viability

Cell viability was assessed using a Cell Counting Kit-8 (CCK-8; Dojindo, Cat#CK04, Japan). Keloid fibroblasts were plated in 96-well plates at a density of 5 × 10^3^ cells per well, with at least five replicate wells for each group, and incubated at 37 °C in a humidified atmosphere containing 5% CO_2_ for 24 h to allow cell attachment prior to treatment. At 48 h post-treatment, 10 μL of CCK-8 reagent was added directly to each well without removing the culture medium, followed by a further 2 h incubation in the dark. The absorbance at 450 nm was then recorded using a microplate reader (BioTek Synergy HTX, United States) to reflect cell metabolic activity. Wells containing only culture medium and CCK-8 solution without cells were used as blanks for background correction.

### 5-Ethynyl-2′-deoxyuridine (EdU) proliferation assay

Cell proliferation was evaluated using a Click-iT™ EdU Imaging Kit (Thermo Fisher, Cat#C10337, United States). After the indicated treatments, keloid fibroblasts were plated on sterile coverslips in 24-well plates. After cell attachment and 24 h of recovery, EdU was added to the culture medium at a final concentration of 10 μM, and cells were incubated for 2 h at 37 °C with 5% CO_2_ to label S-phase cells. The cells were then washed three times with PBS (Gibco, Cat#10010023, United States), fixed in 4% paraformaldehyde (Solarbio, Cat#P1110, China) for 15 min at room temperature, and permeabilized with 0.5% Triton X-100 (Sigma-Aldrich, Cat#T8787, United States) for 20 min. The reaction mixture was prepared according to the kit protocol, and Click labeling was carried out by incubating samples with Apollo staining solution containing fluorescent azide for 30 min in the dark. After washing off excess dye with PBS, nuclei were counterstained with DAPI (Vector Laboratories, Cat#H-1200, United States), and coverslips were mounted. Images were obtained using a confocal laser scanning microscope (Leica TCS SP8, Germany) with identical imaging parameters across all groups. Five randomly selected fields were analyzed per sample. EdU-positive cells and total nuclei were counted using ImageJ software (NIH, Version 1.53t).

### Wound healing assay

Keloid fibroblasts were seeded in 6-well plates and cultured until approximately 90% confluent. A linear scratch was made through the center of the cell monolayer in each well using a sterile 200 μL pipette tip (Axygen, Cat#TF-200-R-S, United States), while maintaining consistent scratching conditions among wells. Detached cells and debris were removed by gently washing the wells two to three times with PBS (Gibco, Cat#10010023, United States). Serum-free DMEM (Gibco, Cat#11965092, United States) was then added to reduce the contribution of cell proliferation to wound closure. Scratch images were captured immediately after wounding (0 h) using an inverted microscope (Olympus CKX53, Japan), and the same regions were photographed again after 24 h. Temperature and CO_2_ levels were kept stable throughout the assay to minimize environmental effects. Wound closure was quantified using ImageJ software (NIH, Version 1.53t) with the Wound Healing Tool plugin. The wound healing rate was calculated using the formula: (initial wound area − wound area at 24 h)/initial wound area × 100%.

### Transwell migration assay

Cell migration was assessed using 24-well Transwell inserts with polycarbonate membranes containing 8 μm pores (Corning, Cat#3422, United States). Before use, the inserts were pre-equilibrated with serum-free medium for 30 min. Keloid fibroblasts were rinsed with PBS, resuspended in serum-free DMEM (Gibco, Cat#11965092, United States), and prepared as a single-cell suspension. A total of 5 × 10^4^ cells in 200 μL medium were added to each upper chamber, while 600 μL DMEM containing 10% FBS (Gibco, Cat#10099141C, United States) was placed in the lower chamber as a chemoattractant. After incubation for 24 h at 37 °C with 5% CO_2_, cells remaining on the upper side of the membrane were gently removed using a cotton swab. The inserts were fixed with 100% methanol (Sinopharm, Cat#100092683, China) for 15 min at room temperature, washed with PBS, and stained with 0.5% crystal violet solution (Solarbio, Cat#G1063, China) for 20 min. After excess dye was removed under running water, the membranes were allowed to air-dry. Migrated cells attached to the lower membrane surface were observed under an inverted microscope (Olympus CKX53, Japan), and five random fields were counted for each sample.

### Western blot (WB)

Total protein was isolated from cells or tissues following the indicated treatments using RIPA lysis buffer (Beyotime, Cat#P0013B, China) containing protease inhibitor cocktail (Beyotime, Cat#ST506, China). Samples were lysed for 30 min and then centrifuged at 12,000 × g for 15 min at 4 °C. The supernatants were collected, and protein concentrations were measured with a BCA Protein Assay Kit (Thermo Fisher, Cat#23225, United States). After normalization, equal amounts of protein were loaded for electrophoresis. Briefly, 30 μg protein from each sample was separated on 10% SDS-PAGE gels (Epizyme, Cat#PG112, China) and transferred onto PVDF membranes (Millipore, Cat#IPVH00010, United States). Membranes were blocked with 5% nonfat milk (Bio-Rad, Cat#1706404, United States) for 1 h at room temperature, followed by overnight incubation at 4 °C with primary antibodies against IL-6 (Abcam, Cat#ab233706, United Kingdom; 1:1,000), IL-8 (R&D Systems, Cat#MAB208, United States; 1:1,000), α-SMA (Abcam, Cat#ab7817, United Kingdom; 1:500), LOX (Abcam, Cat#ab31238, United Kingdom; 1:200), LOXL2 (R&D Systems, Cat#MAB2639, United States; 1:200), COL1A1 (Abcam, Cat#ab34710, United Kingdom; 1:1,000), COL3A1 (Abcam, Cat#ab6310, United Kingdom; 1:1,000), and GAPDH (Cell Signaling Technology, Cat#5174, United States; 1:3,000). On the following day, membranes were washed three times with PBS-T for 10 min each and incubated with the corresponding HRP-conjugated secondary antibodies (Cell Signaling Technology, Cat#7076 or Cat#7074, United States; 1:5,000) for 1 h at room temperature. Protein signals were detected using ECL reagent (Thermo Fisher, Cat#32106, United States). Images were acquired with a Bio-Rad ChemiDoc XRS + imaging system, and band densities were analyzed using Image Lab software (Bio-Rad, Version 6.1.0).

### Reverse transcription-quantitative polymerase chain reaction (RT-qPCR)

Total RNA was isolated from cells using TRIzol reagent (Invitrogen, Cat#15596018, United States). RNA purity and concentration were measured using a NanoDrop 2000 spectrophotometer (Thermo Fisher, United States), and only samples with an A260/280 ratio of 1.8–2.0 were used for downstream analysis. For reverse transcription, 1 μg of total RNA was converted into cDNA using the PrimeScript RT Reagent Kit (Takara, Cat#RR047A, Japan) on a GeneAmp PCR system (Applied Biosystems, United States). Quantitative PCR was carried out with TB Green Premix Ex Taq II (Takara, Cat#RR820A, Japan) on a QuantStudio 6 Flex Real-Time PCR System (Applied Biosystems, United States), with each sample tested in triplicate. The cycling conditions were 95 °C for 30 s, followed by 40 cycles of 95 °C for 5 s and 60 °C for 34 s. Primers were synthesized by TSINGKE (China).

The primer sequences were as follows: *COL1A1*, AGT​GGT​TTG​GAT​GGT​GCC​AA (forward) and GCA​CCA​TCA​TTT​CCA​CGA​GC (reverse); *COL3A1*, AAA​GGC​GAA​GAT​GGC​AAG​GA (forward) and TCC​TGG​GAT​GCC​ATT​TGG​TC (reverse); *LOX*, AGG​GTG​AGG​AGT​AAG​GGA​CC (forward) and AGA​CCT​AAA​CGT​CAG​CAG​GC (reverse); *LOXL2*, GTG​AGC​TCA​GAC​TTG​GTG​CT (forward) and CAA​GGG​CCC​AGA​TGT​CCA​AT (reverse); *IL6*, CCA​CCG​GGA​ACG​AAA​GAG​AA (forward) and GAG​AAG​GCA​ACT​GGA​CCG​AA (reverse); *IL8*, CTC​CAA​ACC​TTT​CCA​CCC​CA (forward) and TTC​TCA​GCC​CTC​TTC​AAA​AAC​T (reverse); *TP53*, AAG​TCT​AGA​GCC​ACC​GTC​CA (forward) and GAC​GCT​AGG​ATC​TGA​CTG​CG (reverse); *CDKN1A*, AGT​CAG​TTC​CTT​GTG​GAG​CC (forward) and GCA​TGG​GTT​CTG​ACG​GAC​AT (reverse); and *GAPDH*, AAT​GGG​CAG​CCG​TTA​GGA​AA (forward) and GCG​CCC​AAT​ACG​ACC​AAA​TC (reverse). Relative gene expression was calculated using the 2^−ΔΔCT^ method and normalized to *GAPDH* as the internal control.

### Enzyme-linked immunosorbent assay (ELISA)

After the indicated treatments, culture supernatants were harvested and centrifuged at 300 *g* for 10 min at 4 °C to eliminate cell debris. The cleared supernatants were aliquoted and stored at −80 °C before detection. IL-6 and IL-8 levels were determined using Human IL-6 ELISA Kit (R&D Systems, Cat#D6050, United States) and Human IL-8 ELISA Kit (R&D Systems, Cat#D8000C, United States), respectively. Before measurement, precoated 96-well plates were brought to room temperature. Standard curves were prepared by serial dilution according to the kit instructions, and samples were diluted at appropriate ratios ranging from 1:2 to 1:5. Standards or samples were added to each well at 100 μL/well and incubated for 2 h at room temperature. After washing three to five times with wash buffer containing PBS and 0.05% Tween-20 (Solarbio, Cat#P1020, China), 100 μL detection antibody working solution was added and incubated for 1 h. The plates were then washed again, followed by incubation with HRP-conjugated streptavidin for 30 min. After further washing, 100 μL TMB substrate solution (Beyotime, Cat#P0209, China) was added to each well and reacted for 10–15 min in the dark. Finally, the reaction was stopped with 50 μL stop solution (2 M H_2_SO_4_; Sigma-Aldrich, Cat#339741, United States). Absorbance was read at 450 nm using a microplate reader (BioTek Synergy H1, United States), with 570 nm used for background correction.

### LOX activity assay

After treatment, cells were harvested and analyzed using a LOX Activity Assay Kit (Abcam, Cat#ab112139, United Kingdom). Cellular proteins were extracted on ice, and protein levels were normalized with a BCA Protein Assay Kit (Thermo Fisher, Cat#23225, United States). Equal amounts of protein were added to each reaction well and mixed with the Amplex Red working solution containing the Amplex Red substrate, HRP, and a LOX-specific substrate. Blank and positive control wells were included in parallel. The total reaction volume was adjusted to 100 μL per well, and the reactions were incubated at 37 °C for 30 min in the dark. Fluorescence intensity was then measured using a multifunctional microplate reader (BioTek Synergy HTX, United States) at excitation/emission wavelengths of 560/590 nm. Each group was analyzed in triplicate, and background fluorescence was subtracted. LOX activity was ultimately expressed as relative fluorescence intensity.

### Soluble collagen fraction assay

Culture supernatants and cell layers were collected separately and preprocessed to isolate soluble and insoluble collagen fractions. Soluble collagen was directly quantified from the supernatant fraction, whereas insoluble collagen was extracted from the cell pellet after chelator-based solubilization and enzymatic digestion. Both fractions were incubated with Sircol dye reagent, allowing collagen to form a precipitable complex with the cationic dye. After incubation for 30 min at room temperature, the precipitates were collected by centrifugation. The supernatants were discarded, and an alkaline dissociation solution was added to release the bound dye. Absorbance was then detected at 562 nm using a microplate reader (BioTek Synergy HTX, United States). Collagen content was calculated based on a standard curve. The ratio of soluble to insoluble collagen was used to indirectly evaluate collagen crosslinking, with a lower ratio indicating increased crosslinking and a higher proportion of insoluble collagen.

### Animal model establishment and intervention

Animal experiments were conducted using healthy male New Zealand White rabbits (*Oryctolagus cuniculus*; 2.5–3.0 kg, 8–10 weeks old; supplied by Beijing Vital River Laboratory Animal Technology Co., Ltd.). Animals were randomly assigned to groups of six and housed under controlled environmental conditions (22 °C ± 2 °C; 12 h light/dark cycle) with free access to food and water. All rabbits were acclimatized for 7 days before the experiments.

The rabbit ear scar model was established using a modified protocol based on previously published methods. Anesthesia was induced by intravenous administration of 3% sodium pentobarbital (Sigma-Aldrich, Cat#P3761, United States) via the marginal ear vein at 1 mL/kg. Full-thickness skin defect surgery was performed under sterile conditions. Four circular full-thickness wounds, each 8 mm in diameter, were created on the dorsal surface of each ear using a biopsy punch (Integra Miltex, Cat#33–31, Germany), extending down to the cartilage layer. Each rabbit, therefore, received a total of eight wounds. Postoperatively, the wounds were irrigated daily with sterile saline (0.9% NaCl; CR Double-Crane, Cat#H20063,963, China) and covered with sterile gauze for 7 consecutive days. To promote hypertrophic scar formation, mechanical stretching and frictional stimulation were applied to the wounds daily for 1 week beginning on postoperative day 7, thereby mimicking a chronic mechanical irritation environment.

On postoperative day 14, animals were randomly assigned to the following treatment groups: (1) control group, receiving no intervention; (2) Sr-90 radiotherapy group, receiving a single local application of 2 Gy to the scar region using a customized Sr-90 applicator; (3) radiotherapy plus p53 inhibition group, receiving intraperitoneal administration of the p53-specific inhibitor Pifithrin-α (Sigma-Aldrich, Cat#P4359, United States) immediately after irradiation at a dose of 2 mg/kg once daily for 5 consecutive days; and (4) radiotherapy plus LOX inhibition group, receiving local subcutaneous injection of β-aminopropionitrile (BAPN; Sigma-Aldrich, Cat#A3134, United States) into the scar area immediately after irradiation at a dose of 100 mg/kg once daily for 5 consecutive days. The same single 2 Gy local exposure was used in the rabbit scar model to maintain consistency with the *in vitro* mechanistic design and to evaluate whether the cellular and matrix-associated changes observed *in vitro* could be reflected at the tissue level. This setting should be interpreted as an exploratory preclinical dosing condition. All animals were euthanized 14 days after intervention, and scar tissues were harvested for subsequent analyses.

### Hematoxylin and eosin (H&E) staining

Collected tissue samples were fixed in 4% paraformaldehyde (Solarbio, Cat#P1110, China) at 4 °C for 24 h. The specimens were then dehydrated through graded ethanol solutions (70%, 80%, 95%, and 100%; Sinopharm Chemical Reagent, China), cleared with xylene (Sinopharm Chemical Reagent, China), and embedded in paraffin using a Leica, E.G.,1150 tissue embedding system. Paraffin blocks were cut into 4 μm sections with a Leica RM2235 microtome and baked at 60 °C for 1 h. Before staining, sections were deparaffinized in xylene and rehydrated through graded ethanol. The sections were then stained with hematoxylin solution (Beyotime, Cat#C0107, China) for 5–8 min, rapidly differentiated in 1% acid alcohol (Solarbio, Cat#G2161, China), and blued, followed by counterstaining with eosin Y solution (Solarbio, Cat#G1100, China) for 1–3 min. Finally, sections were dehydrated, cleared, and mounted with neutral resin mounting medium (Solarbio, Cat#G8590, China). Images were obtained using a Leica DM3000 light microscope.

### Masson’s trichrome staining

Cell samples were grown on coverslips. Animal tissue samples were fixed in 4% paraformaldehyde (Solarbio, Cat#P1110, China) for 24 h, dehydrated through graded ethanol, embedded in paraffin, and serially sectioned at 5 μm. Sections were mounted onto glass slides, deparaffinized, and rehydrated before staining. Masson’s trichrome staining was performed using a commercial kit (Solarbio, Cat#G1340, China). The staining procedure included Weigert’s iron hematoxylin for nuclei, ponceau-acid fuchsin for cytoplasm and muscle fibers, and aniline blue-based cationic dye for collagen fibers. Under this staining condition, collagen appeared blue, muscle tissue appeared red, and nuclei appeared blue-purple. After staining, sections were dehydrated, cleared, and mounted. Images were captured with an Olympus BX53 microscope. Quantitative analysis was conducted using ImageJ software (NIH, Version 1.53t). Collagen-stained regions were selected using the Color Threshold function, and the collagen-positive area percentage was calculated to evaluate collagen deposition.

### Immunohistochemical (IHC) staining

Tissue specimens were fixed in 4% paraformaldehyde (Solarbio, Cat#P1110, China) for 24 h, embedded in paraffin, and sectioned at 5 μm. The sections were then deparaffinized in xylene, rehydrated through graded ethanol, and incubated with 3% H_2_O_2_ (Solarbio, Cat#H885657, China) for 10 min at room temperature to block endogenous peroxidase activity. Antigen retrieval was carried out in 0.01 M sodium citrate buffer by heat-induced epitope retrieval for 15 min. After cooling, sections were washed three times with PBS. After blocking with 5% BSA (Solarbio, Cat#A8020, China) for 1 h, sections were incubated overnight at 4 °C in a humidified chamber with primary antibodies against COL1A1 (Abcam, Cat#ab34710, United Kingdom; 1:300), p53 (Dako/Agilent, Cat#M7001, Denmark; 1:100), p21/CDKN1A (Dako/Agilent, Cat#M7202, Denmark; 1:100), LOX (R&D Systems, Cat#AF7287, United States; 1:100), and LOXL2 (R&D Systems, Cat#AF2639, United States; 1:100). The next day, sections were washed with PBS and incubated with HRP-conjugated secondary antibody (ZSGB-BIO, Cat#PV-6001, China) for 30 min at room temperature. Immunoreactive signals were developed using a DAB substrate kit (ZSGB-BIO, Cat#ZLI-9017, China). Sections were then counterstained with hematoxylin, dehydrated, cleared, and mounted. Cell samples fixed on coverslips were processed using the same antibodies and staining procedure. Images were acquired with an Olympus BX53 microscope. Positive staining was observed as brown-yellow coloration, and DAB-positive regions were considered COL1A1-positive areas. Semiquantitative analysis was performed using ImageJ software (NIH, Version 1.53t) together with the IHC Profiler plugin.

### Total collagen production assay

Total collagen production was measured using a Sircol™ Collagen Assay Kit (Biocolor, Cat#S1000, United Kingdom). Culture supernatants from treated keloid fibroblasts were collected and mixed with an equal volume of Sircol dye reagent. The mixture was incubated for 30 min at room temperature in the dark, allowing the dye to specifically bind soluble collagen and form a collagen-dye precipitate. The precipitates were then collected by centrifugation at 12,000 × g for 10 min. After removal of the supernatant, the collagen-dye pellet was dissolved in alkaline dissociation reagent, and absorbance was measured at 562 nm using a microplate reader (BioTek Synergy HTX, United States).

### Nanoindentation analysis

Nanoindentation measurements were performed using a Piuma Nanoindenter system (Optics11 Life, Netherlands) equipped with a flexible spherical probe (spring constant, 0.5 N/m; probe radius, approximately 10 μm), with data acquisition and analysis conducted using BioSoft software. The test samples consisted of ECM remodeling constructs generated by culturing fibroblasts on collagen scaffolds for 2 weeks. Briefly, type I collagen (Advanced BioMatrix, Cat#5005, United States) was diluted to 2 mg/mL and added to the bottom of 24-well plates to generate gel substrates. After gel formation at 37 °C for 30 min, fibroblasts were seeded at 2 × 10^5^ cells/well and cultured for 14 days in complete medium containing 50 μg/mL ascorbic acid (Sigma-Aldrich, Cat#A5960, United States). The medium was replaced every 2 days. During culture, the cells continuously deposited and remodeled the ECM, giving rise to a collagenous network.

Before mechanical testing, samples were gently rinsed with PBS (Gibco, Cat#10010023, United States) to remove residual medium while preserving ECM hydration, and were then transferred to the nanoindentation platform for *in situ* measurement. For each sample, five distinct regions were selected, and at least 10 indentations were performed per region. Load-indentation depth data were recorded, and stress-strain curves were generated automatically. The elastic modulus and hardness were derived on the basis of the Hertz contact model. All measurements were carried out at room temperature, and the mechanical data were fitted and statistically analyzed using OriginPro software (OriginLab, United States).

### Tensile mechanical testing

Tissue specimens were harvested from the scar regions of treated New Zealand rabbit ears and mounted in a dedicated biomechanical testing fixture. Sample width and thickness were measured and recorded using a precision caliper (Mitutoyo, Cat#500–196–30, Japan) for subsequent stress calculations. Tensile testing was performed using an electronic universal testing machine (Instron 5,943, United States) equipped with a highly sensitive 10 N load cell. Samples were stretched at a constant rate of 0.5 mm/min until failure, and force-displacement data were recorded throughout the test. All experiments were conducted under hydrated conditions, and samples were prewetted with PBS (Gibco, Cat#10010023, United States) before testing to preserve their physiological state. Force-displacement data were converted into stress-strain curves using Bluehill Universal software (Instron, United States).

### Multimodal noninvasive imaging: optical coherence tomography (OCT)

The structural compactness and dermal fibrotic burden of scar tissue were evaluated noninvasively by OCT. Imaging was performed using a skin-dedicated OCT system (VivoSight Dx, Michelson Diagnostics, United Kingdom) with a central wavelength of 1,305 nm, an axial resolution of 7.5 μm, and a lateral resolution of 5 μm. Animals were scanned under light sedation, and the scarred regions of the ear were selected as regions of interest (ROIs). High-resolution cross-sectional images were acquired along the standard depth axis. After image acquisition, hyper-reflective bands within the tissue were automatically identified using the built-*in vivo*Tools analysis software, and the thickness of the dermal fibrotic band was quantified. In addition, the mean optical reflectivity per unit area was extracted as an indicator of collagen packing density and optical anisotropy. For each animal, five fixed points within the scar region were repeatedly scanned. The resulting data were exported and plotted in OriginPro software (OriginLab, United States) for statistical analysis.

### Scanning electron microscopy (SEM)

Scar tissues were harvested from predefined regions and trimmed into approximately 5 mm × 5 mm fragments. The samples were fixed with 2.5% glutaraldehyde (Servicebio, Cat#G1102, China) at 4 °C for 12 h, followed by three washes with PBS (Gibco, Cat#10010023, United States). They were then dehydrated through a graded ethanol series of 30%, 50%, 70%, 80%, 90%, and 100% ethanol (Sinopharm, China), with each concentration applied for 15 min. After dehydration, samples were dried using a critical point dryer (Leica EM CPD300, Germany) and then sputter-coated with platinum using an ion sputter coater (Quorum Q150 R ES, United Kingdom) to enhance conductivity. The specimens were mounted on conductive sample stubs and examined with an SEM (Hitachi SU8100, Japan) at an accelerating voltage of 5–10 kV. High-resolution images were acquired at multiple magnifications, covering the entire scar region, with particular attention to the superficial and mid-depth fiber architecture. Quantitative image analysis was performed using ImageJ software (NIH, Version 1.53t) in combination with the DiameterJ and OrientationJ plugins to determine collagen fiber diameter distribution, fiber density, and alignment coherence.

### Atomic force microscopy (AFM)

Samples were prepared from frozen sections of scar tissue or ECM specimens, mounted onto clean glass slides, and allowed to air-dry naturally before AFM analysis. Measurements were performed using a JPK NanoWizard 4 XP atomic force microscope (Bruker, Germany) equipped with a force spectroscopy module. Quantitative Imaging (QI) mode was selected to enable simultaneous acquisition of topographical and mechanical information. A soft silicon cantilever probe (Bruker, Cat#MLCT, United States) with a spring constant of approximately 0.03 N/m and a tip radius of <20 nm was used. The cantilever spring constant was calibrated before each experiment using the thermal tuning method. Local stiffness was assessed by *in situ* acquisition of force-distance (F-Z) curves, and Young’s modulus was calculated based on the Hertz contact model. The spacing between force mapping points did not exceed 500 nm, and at least 500 valid data points were collected from each sample. Data were processed using JPK Data Processing software and OriginPro (OriginLab, United States).

### Transcriptome sequencing and bioinformatics analysis

Total RNA was isolated from rabbit scar fibroblasts in the control group (n = 3) and radiotherapy group (n = 3) using TRIzol reagent (Invitrogen, Cat#15596018, United States). RNA quality and integrity were examined with an Agilent 2,100 Bioanalyzer (Agilent Technologies, United States), and only samples with RIN values greater than 7.0 were used for library construction. RNA libraries were prepared using the NEBNext Ultra II RNA Library Prep Kit (NEB, Cat#E7770, United States) following the paired-end 150 bp sequencing protocol, and sequencing was performed on an Illumina NovaSeq 6,000 platform (Illumina, United States).

Raw reads were filtered using fastp software (v0.20.0) to remove adaptor contamination and low-quality sequences. The resulting clean reads were mapped to the rabbit reference genome OryCun2.0 with STAR software (v2.7.3a). Gene count matrices were then used for differential expression analysis with DESeq2 (v1.30.0). Differentially expressed genes (DEGs) were identified using the thresholds of |log_2_ fold change| > 1 and false discovery rate (FDR) < 0.05, and volcano plots were generated to visualize the transcriptional alterations.

Functional enrichment analysis was performed using the ClusterProfiler package (v4.2.0) for Gene Ontology (GO) annotation and Kyoto Encyclopedia of Genes and Genomes (KEGG) pathway analysis. Upregulated and downregulated genes were analyzed separately to characterize their enrichment trends across biological process (BP), cellular component (CC), and molecular function (MF) categories, and significantly enriched pathways were visualized using bubble plots. Principal component analysis (PCA) was conducted in the R environment (v4.1.2) based on the normalized expression matrix to assess global transcriptional differences among samples. All bioinformatics analyses were performed in R (v4.1.2).

### Statistical analysis

Data are expressed as mean ± standard deviation (SD). GraphPad Prism 9 (GraphPad Software, United States) and R software (v4.1.2; R Foundation for Statistical Computing, Austria) were used for data analysis and visualization. For comparisons between two groups, an unpaired two-tailed Student’s t-test was applied. Differences among multiple groups were evaluated using one-way analysis of variance (one-way ANOVA). When ANOVA showed significant differences, Tukey’s *post hoc* test was further performed for pairwise comparisons. Correlation analysis was performed using Pearson’s correlation coefficient (*r*) to evaluate the strength of linear associations between variables, and the results were presented as scatter plots with regression lines and confidence intervals. A value of *p* < 0.05 was considered statistically significant. All experiments included at least three independent biological replicates, and technical replicates were included where appropriate to ensure data robustness and statistical reliability. Statistical significance in figures is denoted using the standard notation: *p < 0.05, **p < 0.01, ***p < 0.001, and ****p < 0.0001.

## Results

### Sr-90 superficial radiotherapy is associated with p53-p21 signaling activation and senescence-associated changes in keloid fibroblasts

**FIGURE 1:**
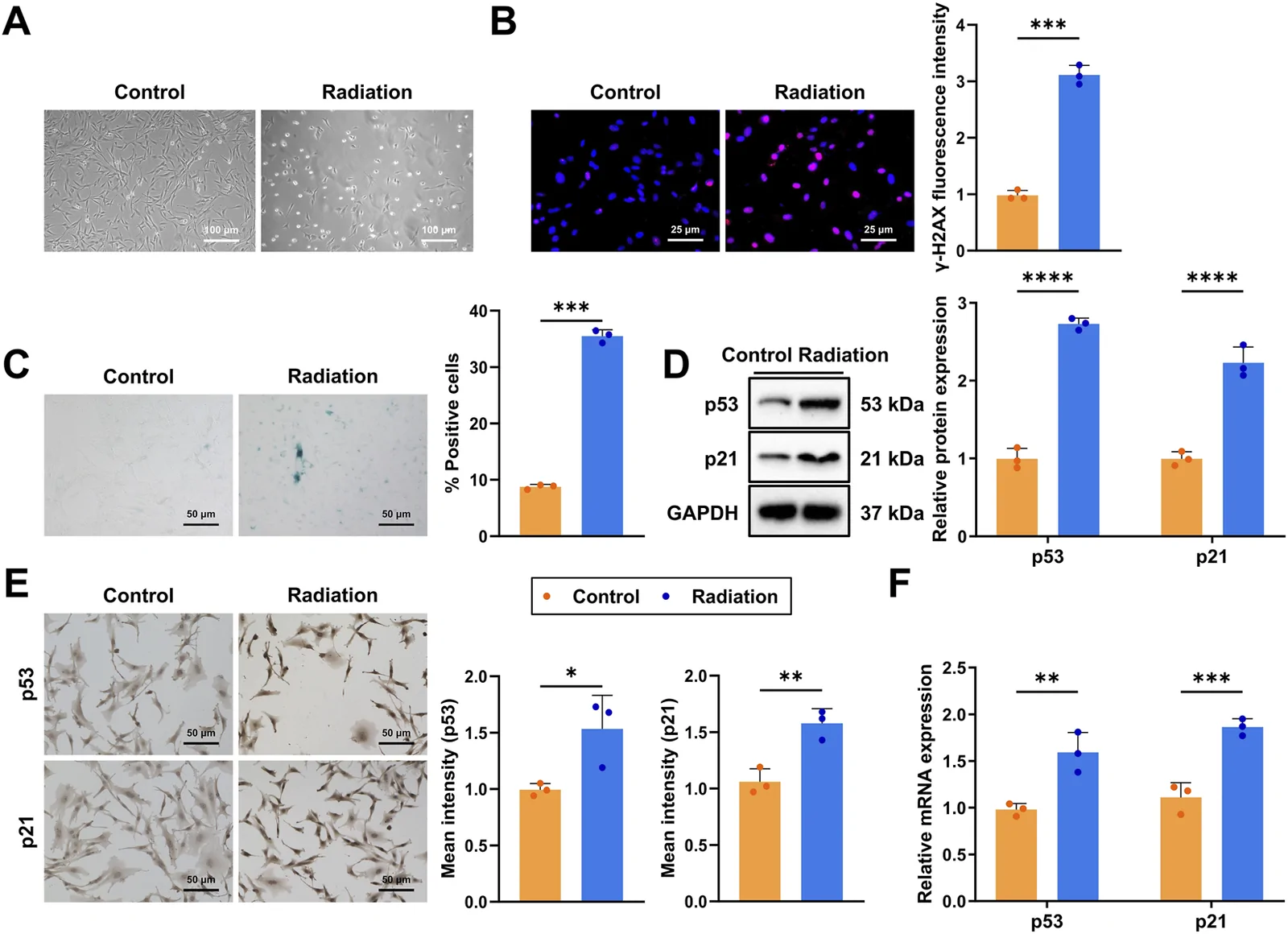
Sr-90 superficial radiotherapy is associated with fibroblast senescence-related changes accompanied by p53-p21 pathway activation. Note **(A)** Representative morphological changes in keloid fibroblasts; scale bar = 100 μm **(B)** IF analysis of nuclear γ-H2AX foci in keloid fibroblasts; scale bar = 25 μm **(C)** SA-β-gal staining showing senescence-associated β-galactosidase positivity in keloid fibroblasts; scale bar = 50 μm **(D)** WB analysis of p53 and p21 protein expression in keloid fibroblasts **(E)** IHC analysis of nuclear p53 and p21 expression in keloid fibroblasts; scale bar = 50 μm **(F)** RT-qPCR analysis of *TP53* and *CDKN1A* mRNA expression in keloid fibroblasts. Cell-based experiments were performed in triplicate. Asterisks indicate comparisons between two groups: *p < 0.05, **p < 0.01, ***p < 0.001, and ****p < 0.0001.

To determine whether Sr-90 superficial radiotherapy is associated with senescence-related changes and DDR activation in keloid fibroblasts, we performed a systematic analysis using an *in vitro* model. Compared with untreated controls, irradiated fibroblasts showed obvious morphological changes, shifting from a spindle-like shape to a flattened and enlarged appearance (Figure 1A). IF staining revealed a pronounced increase in nuclear γ-H2AX foci in the irradiated group, indicating robust activation of the DDR (Figure 1B). Quantitative single-cell fluorescence analysis further verified that γ-H2AX fluorescence intensity was significantly increased in the irradiated group compared with the control group (Figure 1B).

Senescence evaluation showed that SA-β-gal-positive cells were significantly more abundant after irradiation, indicating that Sr-90 exposure was associated with a senescence-like phenotype in keloid fibroblasts (Figure 1C). WB further demonstrated significant upregulation of p53 and p21 protein levels following irradiation (Figure 1D). Consistently, IHC staining showed stronger nuclear p53 and p21 signals in irradiated cells than in controls (Figure 1E). At the mRNA level, RT-qPCR results showed that TP53 and CDKN1A expression was likewise significantly elevated after irradiation (Figure 1F).

Taken together, these findings indicate that Sr-90 superficial radiotherapy markedly activates the γ-H2AX-associated DDR in keloid fibroblasts and enhances p53-p21 signaling, accompanied by conspicuous morphological remodeling and the emergence of senescence-associated phenotypes, consistent with a senescence-like growth-arrested phenotype.

### Sr-90 superficial radiotherapy attenuates the proliferative and migratory capacity of keloid fibroblasts

To clarify how Sr-90 superficial radiotherapy affects the growth behavior of keloid fibroblasts, we conducted a multidimensional assessment focusing on cell viability, migratory capacity, and cytoskeletal organization.

**FIGURE 2:**
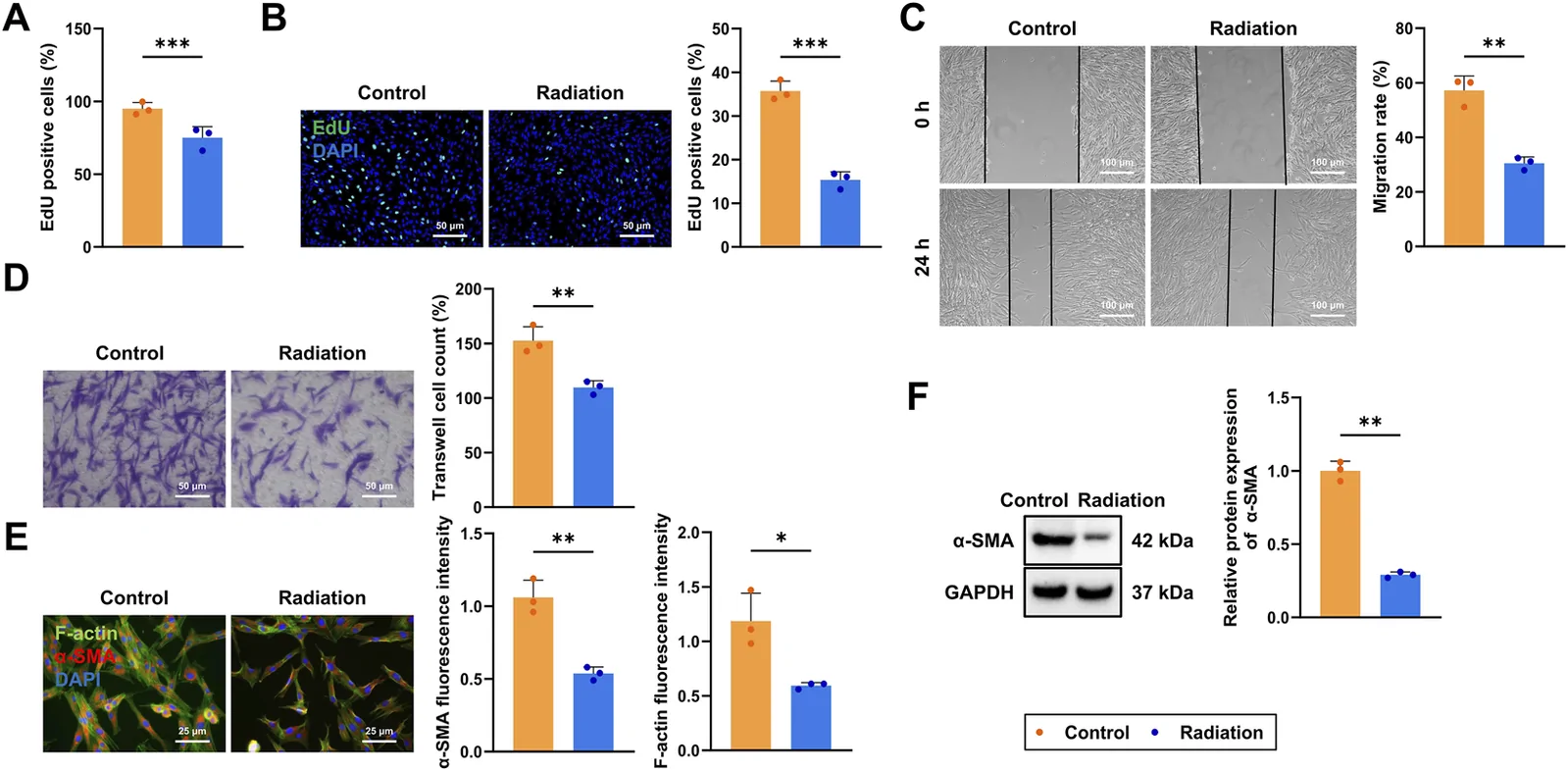
Sr-90 superficial radiotherapy is associated with reduced proliferation, migration, and myofibroblastic features in keloid fibroblasts. Note **(A)** CCK-8 assay of keloid fibroblast viability **(B)** EdU incorporation assay for proliferation of keloid fibroblasts **(C)** Wound healing assay showing the migratory distance of keloid fibroblasts; scale bar = 100 μm **(D)** Transwell assay evaluating the migratory capacity of keloid fibroblasts **(E)** IF analysis of F-actin fiber architecture and α-SMA expression in keloid fibroblasts; scale bar = 50 μm **(F)** WB analysis of α-SMA protein expression in keloid fibroblasts. Cell-based experiments were performed in triplicate. Asterisks indicate comparisons between two groups: *p < 0.05, **p < 0.01, and ***p < 0.001.

CCK-8 results indicated that irradiation significantly decreased the viability of keloid fibroblasts compared with the control group (Figure 2A). EdU incorporation analysis further demonstrated a marked decrease in proliferative activity after irradiation (Figure 2B). Evaluation of cell motility showed that wound closure was significantly impaired in the irradiated group in the scratch assay (Figure 2C). Similarly, Transwell assays revealed a significant reduction in the number of cells migrating through the membrane following irradiation (Figure 2D).

IF analysis of cytoskeletal architecture showed that, relative to the control group, irradiated cells displayed pronounced disorganization of filamentous actin (F-actin) fibers, diminished peripheral tension, and reduced α-SMA fluorescence intensity (Figure 2E). Consistent with these observations, WB confirmed that α-SMA protein expression was significantly decreased after irradiation (Figure 2F).

Collectively, these results demonstrate that Sr-90 superficial radiotherapy significantly suppresses the viability, proliferation, and migration of keloid fibroblasts *in vitro*, while also weakening cytoskeletal tension and destabilizing the myofibroblastic phenotype.

### Sr-90 superficial radiotherapy is accompanied by a senescence-associated secretory phenotype (SASP)-like inflammatory response

**FIGURE 3:**
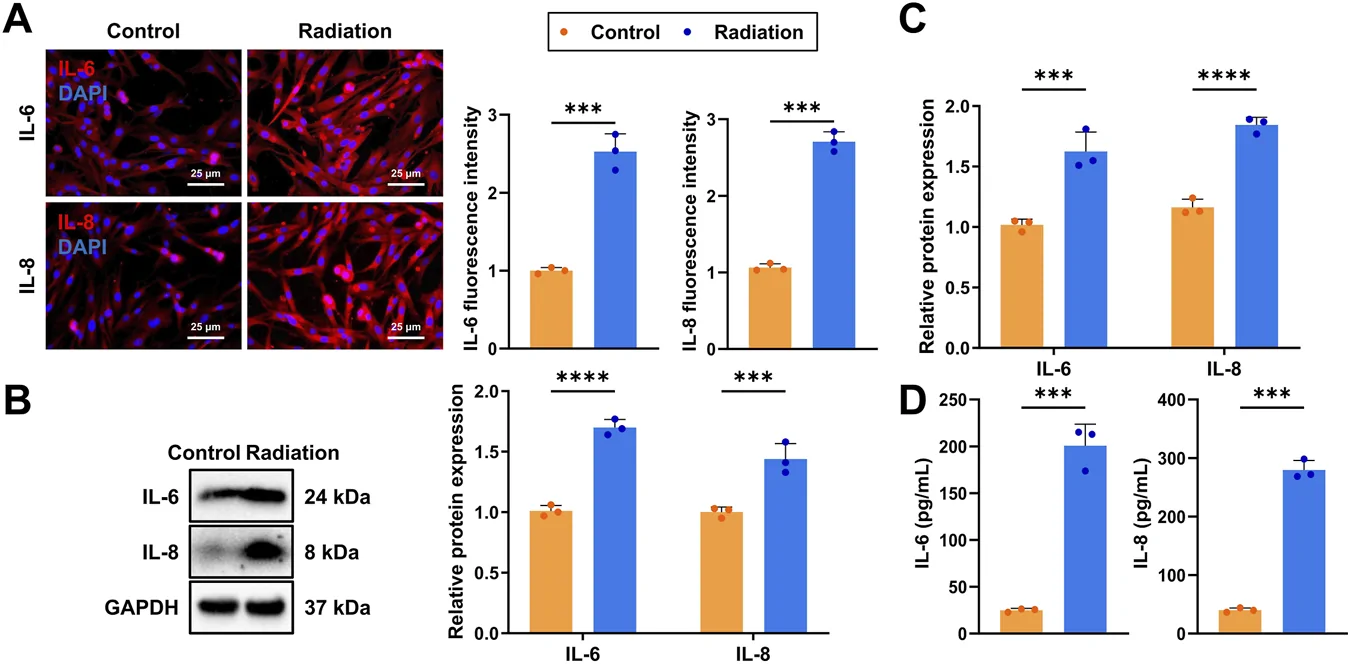
Sr-90 superficial radiotherapy is accompanied by a SASP-like increase in IL-6/IL-8 expression and secretion. Note **(A)** IF analysis of the intracellular localization and fluorescence intensity of IL-6 and IL-8 in keloid fibroblasts; scale bar = 25 μm **(B)** WB analysis of IL-6 and IL-8 protein expression in keloid fibroblasts **(C)** RT-qPCR analysis of *IL6* and *IL8* mRNA expression in keloid fibroblasts **(D)** ELISA analysis of IL-6 and IL-8 secretion in the culture supernatant of keloid fibroblasts. Cell-based experiments were performed in triplicate. Asterisks indicate comparisons between two groups: ***p < 0.001, ****p < 0.0001.

To determine whether senescence-associated changes after Sr-90 superficial radiotherapy are accompanied by a SASP-like inflammatory response in keloid fibroblasts, we examined the expression and secretion profiles of key inflammatory mediators. IF staining showed substantially stronger IL-6 and IL-8 signals in irradiated cells than in untreated controls (Figure 3A).

WB further demonstrated significant upregulation of IL-6 and IL-8 protein levels following irradiation (Figure 3B). Consistent with these findings, RT-qPCR revealed significantly increased mRNA expression of *IL6* and *IL8* in irradiated cells relative to controls (Figure 3C). ELISA analysis also demonstrated enhanced secretion of IL-6 and IL-8 following irradiation (Figure 3D).

Together, these findings indicate that Sr-90 superficial radiotherapy markedly enhances both the expression and secretion of IL-6 and IL-8 in keloid fibroblasts, consistent with a SASP-like inflammatory program.

### Sr-90 superficial radiotherapy markedly downregulates LOX/LOXL2 expression and reduces enzymatic activity

To explore the molecular basis by which Sr-90 superficial radiotherapy improves scar architecture, we focused on its effects on the key collagen crosslinking enzymes LOX and its homolog LOXL2.

**FIGURE 4:**
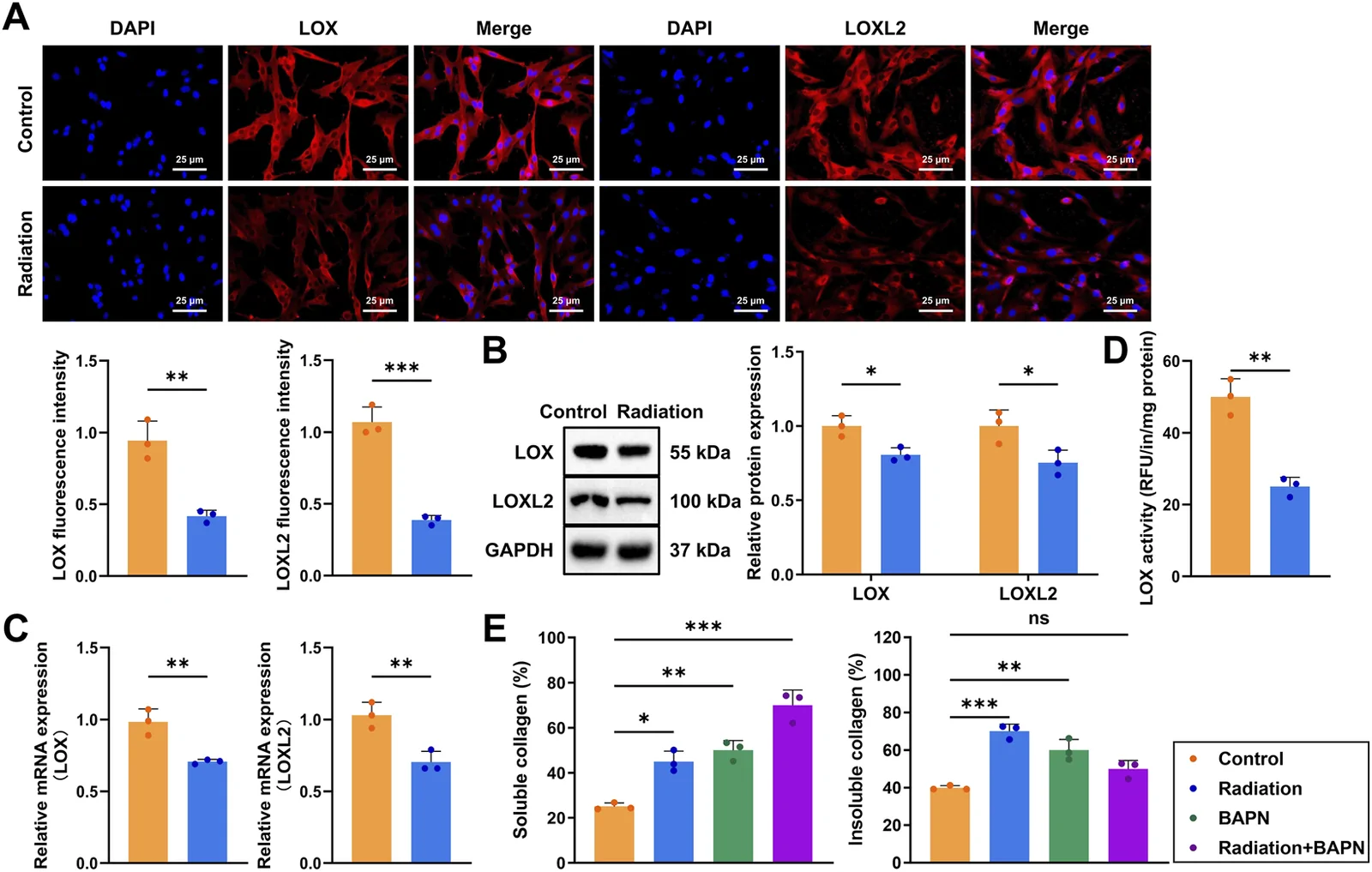
Sr-90 superficial radiotherapy downregulates LOX/LOXL2 expression and reduces LOX-associated enzymatic activity. Note **(A)** IF analysis of the cellular localization and fluorescence intensity of LOX and LOXL2 in fibroblasts; scale bar = 25 μm **(B)** WB analysis of LOX and LOXL2 protein expression in fibroblasts **(C)** RT-qPCR analysis of *LOX* and *LOXL2* mRNA expression in fibroblasts **(D)** Total LOX enzymatic activity in fibroblasts measured by the Amplex Red assay **(E)** Soluble collagen extraction assay showing changes in the ratio of soluble to insoluble collagen in fibroblasts. Cell-based experiments were performed in triplicate. Asterisks indicate comparisons between two groups: *p < 0.05, **p < 0.01, and ***p < 0.001.

In cultured fibroblasts, IF staining showed that LOX and LOXL2 were predominantly distributed in the ECM and the pericellular membrane-associated region. Compared with the control group, irradiated cells exhibited significantly reduced signal intensity for both proteins, together with a visibly sparser fibrillar distribution pattern (Figure 4A). WB confirmed these changes, demonstrating significant decreases in LOX and LOXL2 protein abundance after irradiation (Figure 4B). At the mRNA level, RT-qPCR analysis demonstrated that *LOX* and *LOXL2* expression was markedly decreased in irradiated cells compared with the control group (Figure 4C). To assess overall LOX-associated enzymatic activity, total enzymatic activity was measured using the Amplex Red assay. LOX activity was significantly decreased following irradiation (Figure 4D), consistent with reduced LOX/LOXL2-associated matrix crosslinking activity.

To further evaluate the combined effects of LOX inhibition and radiotherapy, the soluble collagen fraction was additionally quantified. Relative to the control group, the proportion of soluble collagen was significantly elevated in the BAPN-treated group, and a comparable increase was also observed following irradiation. Notably, the combination treatment produced a further significant increase in soluble collagen relative to either monotherapy group, accompanied by a corresponding decrease in the insoluble collagen fraction (Figure 4E). These results suggest that BAPN and radiotherapy produced a stronger combined effect on soluble and insoluble collagen fractions than either treatment alone.

Taken together, Sr-90 superficial radiotherapy significantly downregulates LOX/LOXL2 expression and attenuates LOX enzymatic activity, which may contribute to reduced collagen crosslinking-related matrix stabilization and improved tissue mechanical properties. These findings identify reduced LOX/LOXL2 expression and activity as a matrix-associated component of radiotherapy-related scar remodeling.

### Enhanced antifibrotic effects of Sr-90 superficial radiotherapy combined with a LOX inhibitor

To evaluate the enhanced combined effects of Sr-90 superficial radiotherapy and LOX inhibition, we performed a systematic analysis in human scar fibroblasts treated with radiotherapy alone, BAPN alone, or the combined regimen.

**FIGURE 5:**
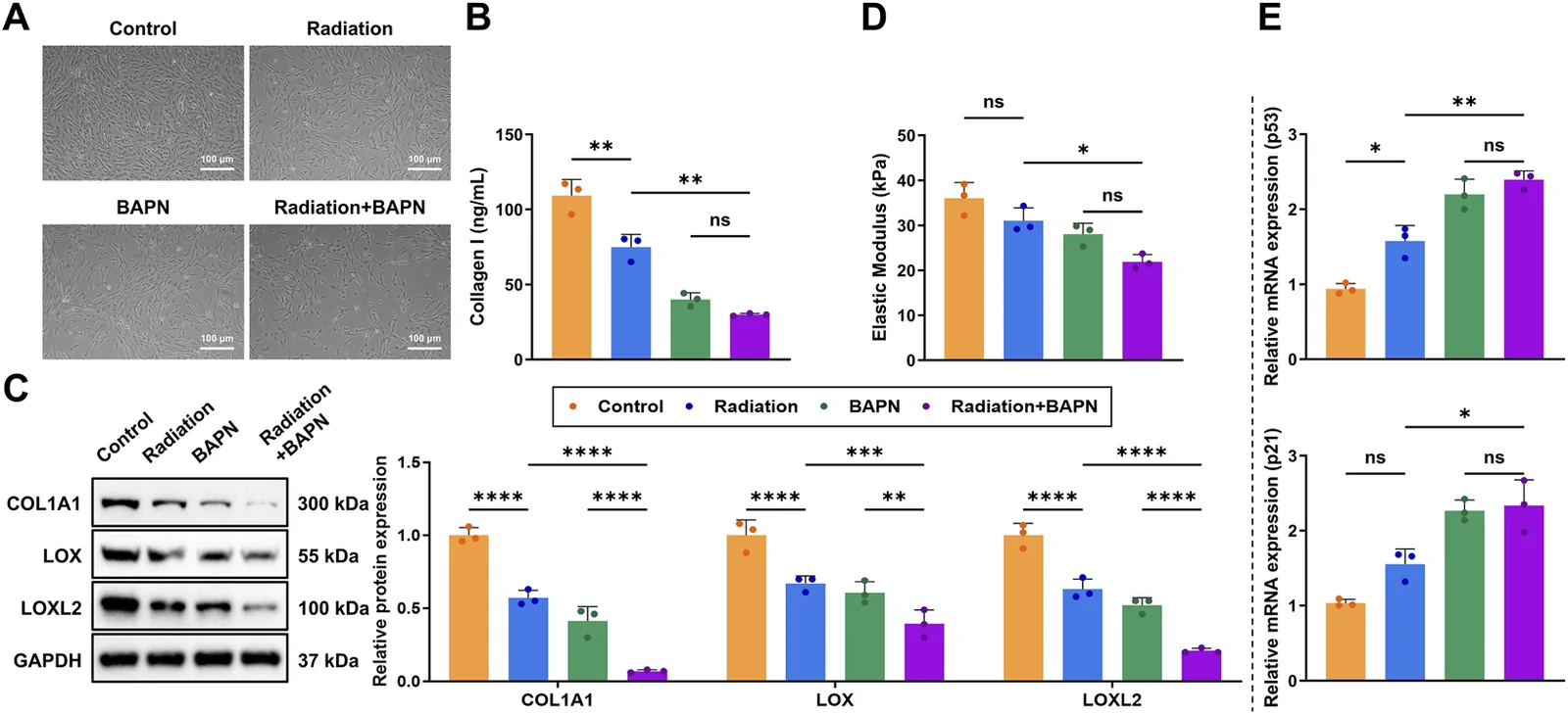
The combination of Sr-90 superficial radiotherapy and LOX inhibition produces greater changes in collagen crosslinking-related indices and mechanical properties than either treatment alone. Note **(A)** Representative light microscopy images showing the morphology of scar fibroblasts; scale bar = 100 μm **(B)** ELISA analysis of collagen secretion in the culture supernatant of scar fibroblasts **(C)** WB analysis of COL1A1, LOX, and LOXL2 protein expression in scar fibroblasts **(D)** Nanoindentation analysis of changes in the elastic modulus of fibroblast-derived matrix constructs **(E)** RT-qPCR analysis of *TP53* and *CDKN1A* gene expression in scar fibroblasts. Cell-based experiments were performed in triplicate. Asterisks indicate comparisons between two groups: *p < 0.05, **p < 0.01, ***p < 0.001, and ****p < 0.0001.

Morphological examination showed that, compared with the control group, cells in both the radiotherapy and BAPN groups became more flattened and dispersed. This phenotypic shift was even more pronounced in the combination group than in either monotherapy group (Figure 5A).

Quantification of collagen secretion showed that both radiotherapy and BAPN alone significantly reduced collagen production relative to the control group, whereas the combination treatment led to a further significant decrease beyond that observed with either single treatment (Figure 5B). WB was then used to assess proteins involved in collagen metabolism. Compared with the control group, COL1A1, LOX, and LOXL2 protein levels were significantly reduced in both the radiotherapy and BAPN groups. These reductions were further accentuated in the combination group relative to either single-treatment group (Figure 5C). Nanoindentation-based mechanical analysis showed that the elastic modulus was significantly decreased in both the radiotherapy and BAPN groups compared with the control group. The combination treatment induced a further decline in elastic modulus, indicating lower matrix stiffness and a more compliant mechanical phenotype (Figure 5D). RT-qPCR analysis revealed that, compared with the control group, the expression of *TP53* and *CDKN1A* was significantly increased in the radiotherapy group; moreover, these transcripts were further upregulated in the combination group relative to radiotherapy alone (Figure 5E).

Collectively, these results demonstrate that combined Sr-90 radiotherapy and LOX inhibition produced a stronger antifibrotic effect than either treatment alone in scar fibroblasts. Combined treatment was accompanied by further increases in TP53 and CDKN1A mRNA levels, greater reductions in collagen-related proteins and collagen deposition, and lower matrix stiffness.

### Sr-90 superficial radiotherapy attenuates matrix accumulation and collagen metabolic activity in fibroblasts

To determine whether Sr-90 superficial radiotherapy-related changes in scar architecture are accompanied by reduced collagen synthesis in fibroblasts, we systematically examined collagen matrix deposition and the expression of collagen-related genes.

**FIGURE 6:**
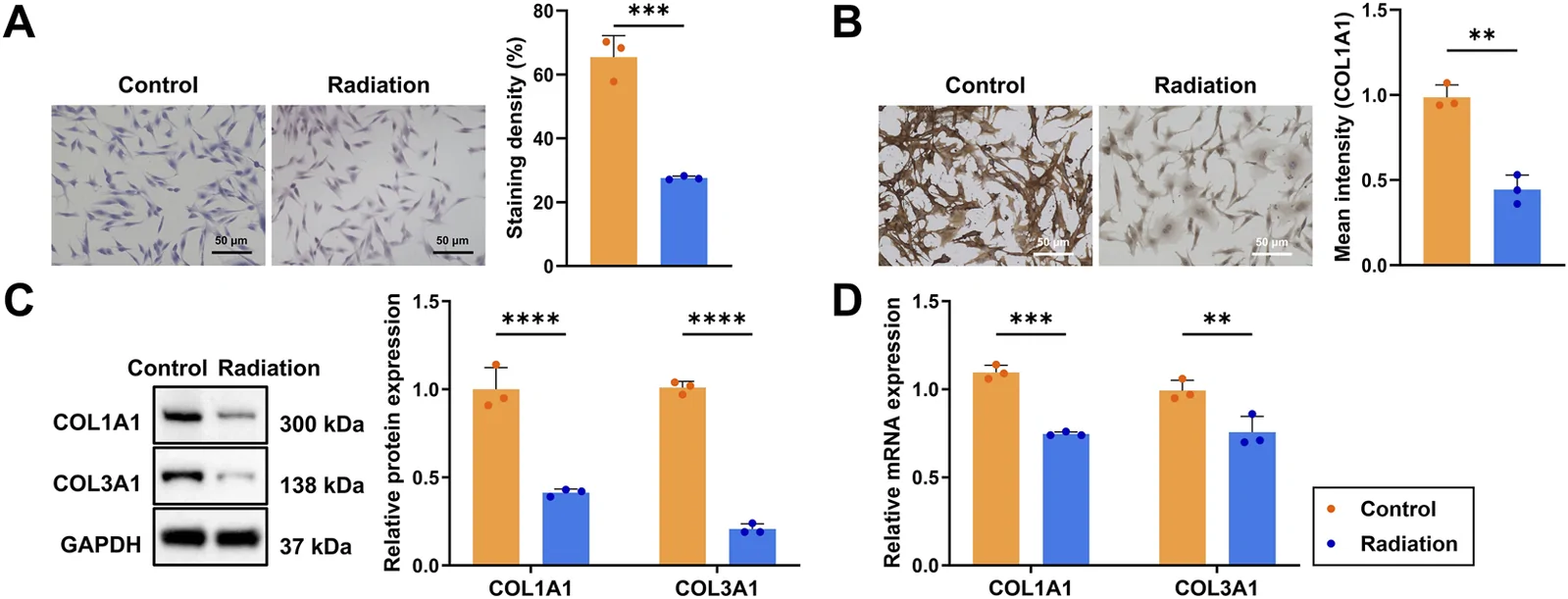
Sr-90 superficial radiotherapy is associated with reduced collagen synthesis and matrix accumulation in fibroblast-derived matrix. Note **(A)** Masson’s trichrome staining showing collagen deposition and fiber arrangement in fibroblast-derived matrix; scale bar = 50 μm **(B)** IHC analysis of COL1A1 expression and the positive staining area in the fibroblast-derived ECM; scale bar = 50 μm **(C)** WB analysis of COL1A1 and COL3A1 protein expression in fibroblasts **(D)** RT-qPCR analysis of *COL1A1* and *COL3A1* mRNA expression in fibroblasts. Cell-based experiments were performed in triplicate. Asterisks indicate comparisons between two groups: *p < 0.05, **p < 0.01, ***p < 0.001, and ****p < 0.0001.

Masson’s trichrome staining showed that, compared with the control group, collagen fibers in the matrix produced by irradiated fibroblasts shifted from a densely disordered arrangement to a looser and more parallel pattern, accompanied by a marked reduction in collagen deposition (Figure 6A). IHC staining further demonstrated that both the staining intensity and positive area of COL1A1 in the fibroblast-derived ECM were significantly reduced after radiotherapy (Figure 6B), indicating diminished collagen-producing capacity. WB confirmed that the protein levels of COL1A1 and COL3A1 were both significantly decreased in the irradiated group relative to the control group (Figure 6C). Consistently, RT-qPCR analysis showed significant downregulation of *COL1A1* and *COL3A1* mRNA expression following irradiation (Figure 6D).

Taken together, these results show that Sr-90 superficial radiotherapy markedly inhibits collagen synthesis and deposition at the cellular level, thereby alleviating excessive matrix accumulation.

### Sr-90 superficial radiotherapy improves scar architecture *in vivo*

**FIGURE 7:**
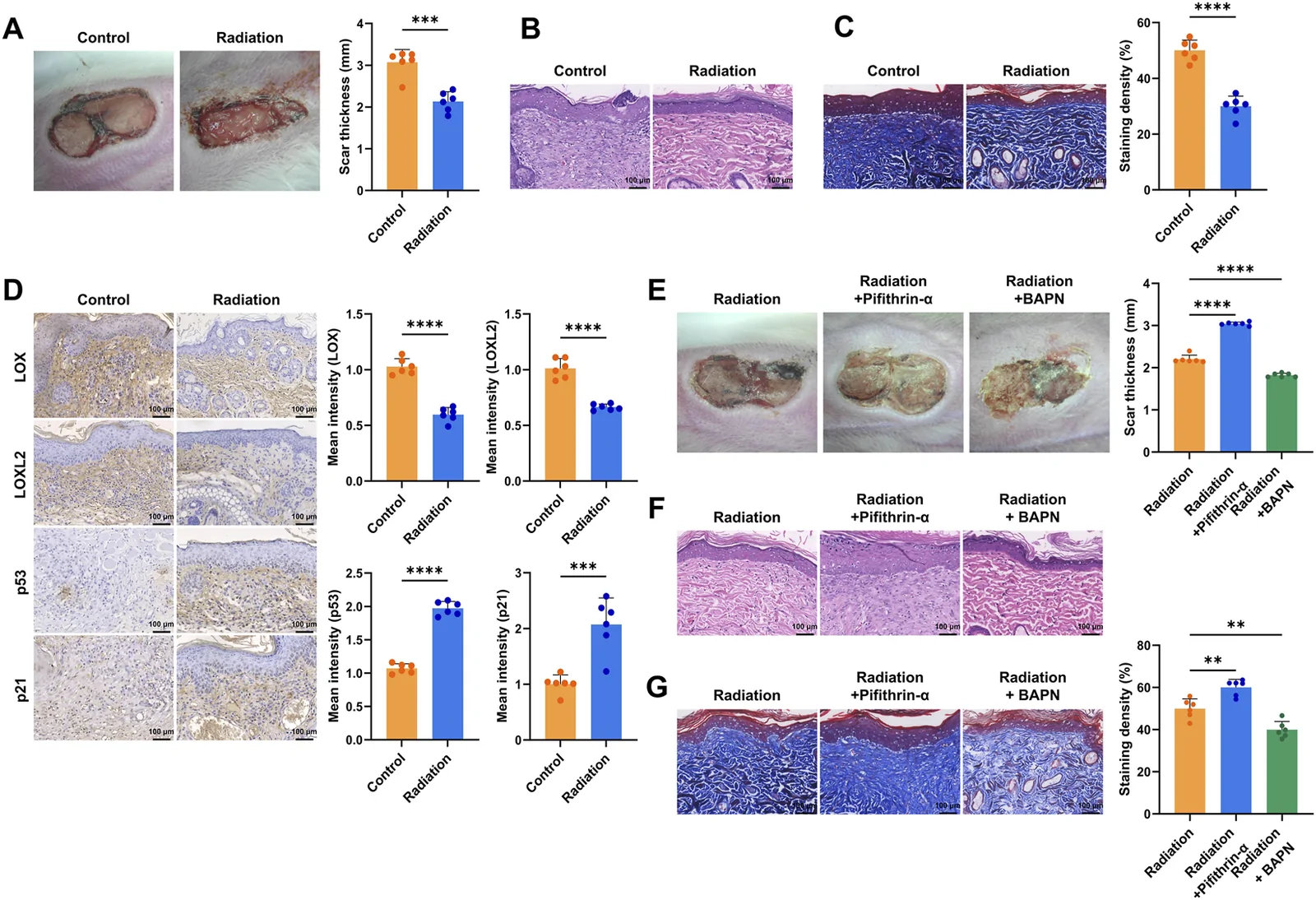
Sr-90 superficial radiotherapy is associated with reduced scar thickness and improved tissue mechanical properties in the rabbit ear scar model. Note **(A)** Gross morphological evaluation of the effects of radiotherapy on scar appearance in the rabbit ear scar model **(B)** H&E staining showing overall structural changes in scar tissue; scale bar = 100 μm **(C)** Masson’s trichrome staining showing collagen distribution and the degree of fibrosis **(D)** IHC analysis of p53, p21, LOX, and LOXL2 expression; scale bar = 100 μm **(E)** Evaluation of the effects of Pifithrin-α and BAPN on radiotherapy efficacy following combination treatment **(F)** H&E staining assessing the effects of combination treatment on collagen architecture; scale bar = 100 μm **(G)** Masson’s trichrome staining, analyzing the regulation of fibrotic severity by combination treatment; scale bar = 100 μm. Six animals were included in each group. Asterisks indicate comparisons between two groups: **p < 0.01, ****p < 0.0001.

To evaluate the antiscarring efficacy of Sr-90 superficial radiotherapy *in vivo*, we established a New Zealand rabbit ear scar model for systematic validation. Compared with the control group, the radiotherapy group exhibited a significant reduction in scar volume, a smoother surface contour, and decreased scar thickness (Figure 7A). Histological staining further substantiated this structural remodeling. H&E staining as well as Masson’s trichrome staining showed that, relative to the control group, dermal collagen fibers in the radiotherapy group were transformed from densely packed bundles into a looser, undulating architecture, with a marked decrease in collagen staining density (Figures 7B,C). IHC analysis further revealed significant upregulation of p53 and p21 staining intensity, accompanied by significant downregulation of LOX and LOXL2 staining, in the radiotherapy group compared with the control group (Figure 7D).

To verify the functional roles of the key pathways, the radiotherapy regimen was combined separately with the p53 inhibitor Pifithrin-α and the LOX inhibitor BAPN. Morphological assessment showed that, compared with radiotherapy alone, Pifithrin-α treatment substantially blunted the reductions in scar thickness and the improvement in tissue elasticity, whereas BAPN further enhanced the structural benefits induced by radiotherapy (Figure 7E). H&E staining and Masson’s trichrome staining further confirmed these findings: compared with the radiotherapy group, collagen fibers in the Pifithrin-α group reacquired a densely bundled arrangement, whereas those in the BAPN group became even looser, with a further reduction in fibrotic severity (Figures 7F,G).

Overall, the *in vivo* results were generally consistent with the *in vitro* findings. Sr-90 superficial radiotherapy was associated with improved scar architecture and mechanical properties, together with enhanced p53/p21 signaling and reduced LOX-related molecular signals.

### Sr-90 superficial radiotherapy remodels the mechanical properties of scar tissue and restores skin elasticity

To assess the effects of Sr-90 superficial radiotherapy on the biomechanical performance of scar tissue, we performed a systematic analysis of elasticity, hardness, and microstructural features in the scar region.

**FIGURE 8:**
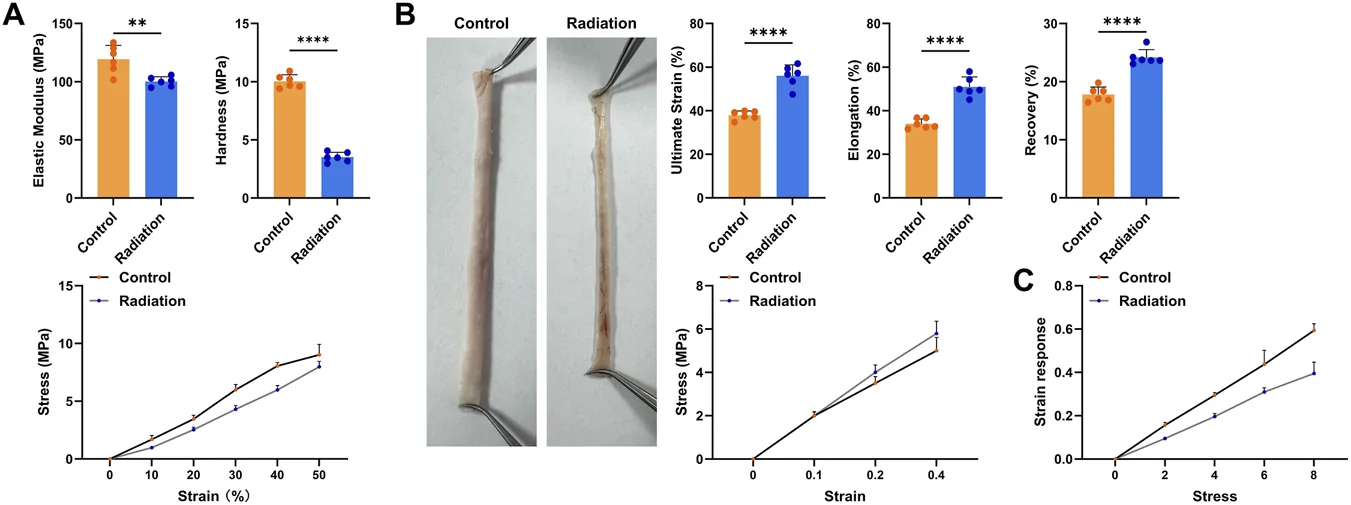
Sr-90 superficial radiotherapy is associated with improved mechanical properties and elastic recovery in scar tissue. Note **(A)** Nanoindentation analysis of scar tissue showing changes in elastic modulus, hardness, and stress-strain characteristics **(B)** Tensile testing of scar tissue showing ultimate strain, extensibility, and recovery capacity; scale bar = 2 mm **(C)** Biomechanical response analysis showing strain responsiveness of scar tissue in the low-stress region. Six animals were included in each group. Asterisks indicate comparisons between two groups: *p < 0.05, **p < 0.01, and ****p < 0.0001.

Nanoindentation results showed that irradiated scar tissues had significantly lower elastic modulus and hardness than controls, while the stress–strain curves suggested improved deformability (Figure 8A). Tensile testing further revealed that ultimate strain and extensibility were markedly increased after radiotherapy, with recoil capacity and energy absorption also showing upward trends (Figure 8B). Analysis of skin mechanical response curves further showed that strain responsiveness in the low-stress region was significantly enhanced after radiotherapy, shifting the overall mechanical profile toward lower stiffness and greater deformability (Figure 8C).

Collectively, these findings indicate that Sr-90 superficial radiotherapy substantially improves the biomechanical behavior of scar tissue at the tissue level, increasing elasticity while reducing hardness, in parallel with ECM loosening and fibrous reorganization. Together, these changes shift scar tissue from a high-stiffness state toward a more compliant mechanical phenotype.

### Sr-90 superficial radiotherapy remodels the microstructural collagen architecture and spatial organization of scar tissue

To determine how Sr-90 superficial radiotherapy influences collagen fiber configuration and tissue organization at the ultrastructural level, we systematically characterized collagen morphology, orientation, and local mechanical properties.

**FIGURE 9:**
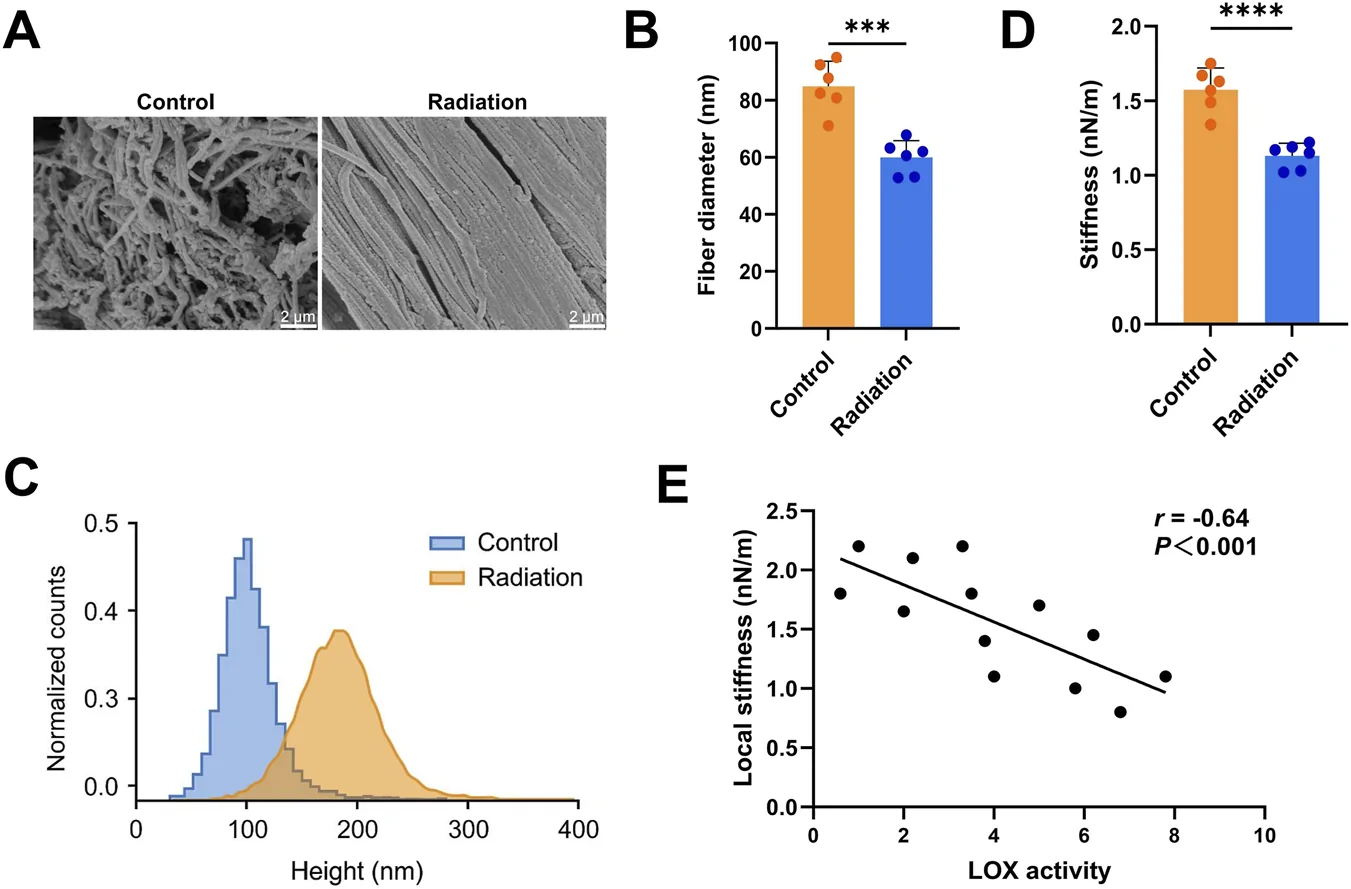
Sr-90 superficial radiotherapy is associated with remodeling of collagen microarchitecture and fiber orientation in scar tissue. Note **(A)** SEM images showing the overall morphology and organizational features of collagen fibers in scar tissue; scale bar = 100 μm **(B)** Quantitative SEM-based analysis of collagen fiber diameter in scar tissue **(C)** AFM height distribution curves showing the distribution characteristics of collagen fiber surface height in scar tissue. Height refers to the AFM-measured surface topography rather than fiber diameter and was used to characterize surface roughness and height distribution **(D)** AFM force spectroscopy analysis of the local stiffness of collagen fibers in scar tissue **(E)** Correlation analysis between local collagen fiber stiffness and collagen crosslinking enzyme activity. Six animals were included in each group. Asterisks indicate comparisons between two groups: *p < 0.05, ***p < 0.001, and ****p < 0.0001.

SEM revealed a marked increase in collagen fiber regularity in the radiotherapy group compared with the control group. In the control group, collagen fibers were thick, disordered, and extensively interwoven, whereas irradiated scars displayed finer fibers with more consistent orientation and a more prominent parallel bundle-like arrangement (Figure 9A). Quantitative analysis further showed a significant reduction in fiber diameter following radiotherapy (Figure 9B).

Height distribution profiles exhibited a distinctly multimodal pattern in the control group but converged into a unimodal distribution in the radiotherapy group, indicating that collagen microarchitecture became substantially more homogeneous after treatment (Figure 9C). AFM-based force spectroscopy further showed that the local stiffness of collagen fibers was significantly reduced in irradiated tissue relative to controls (Figure 9D). Correlation analysis identified a significant association between local fiber stiffness and collagen crosslinking enzyme activity (Figure 9E), linking variation in microscale stiffness to variation in crosslinking-related enzyme activity.

Collectively, these findings show that Sr-90 superficial radiotherapy is associated with marked reorganization of the collagen fiber network at the ultrastructural level, transforming a dense and disordered matrix into a more uniform, ordered, and compliant architecture. These ultrastructural changes are consistent with scar softening and improved elasticity, and support a role for radiotherapy in modulating ECM organization.

### Sr-90 superficial radiotherapy is associated with transcriptomic changes in transcriptional regulatory and ECM-associated pathways.

**FIGURE 10:**
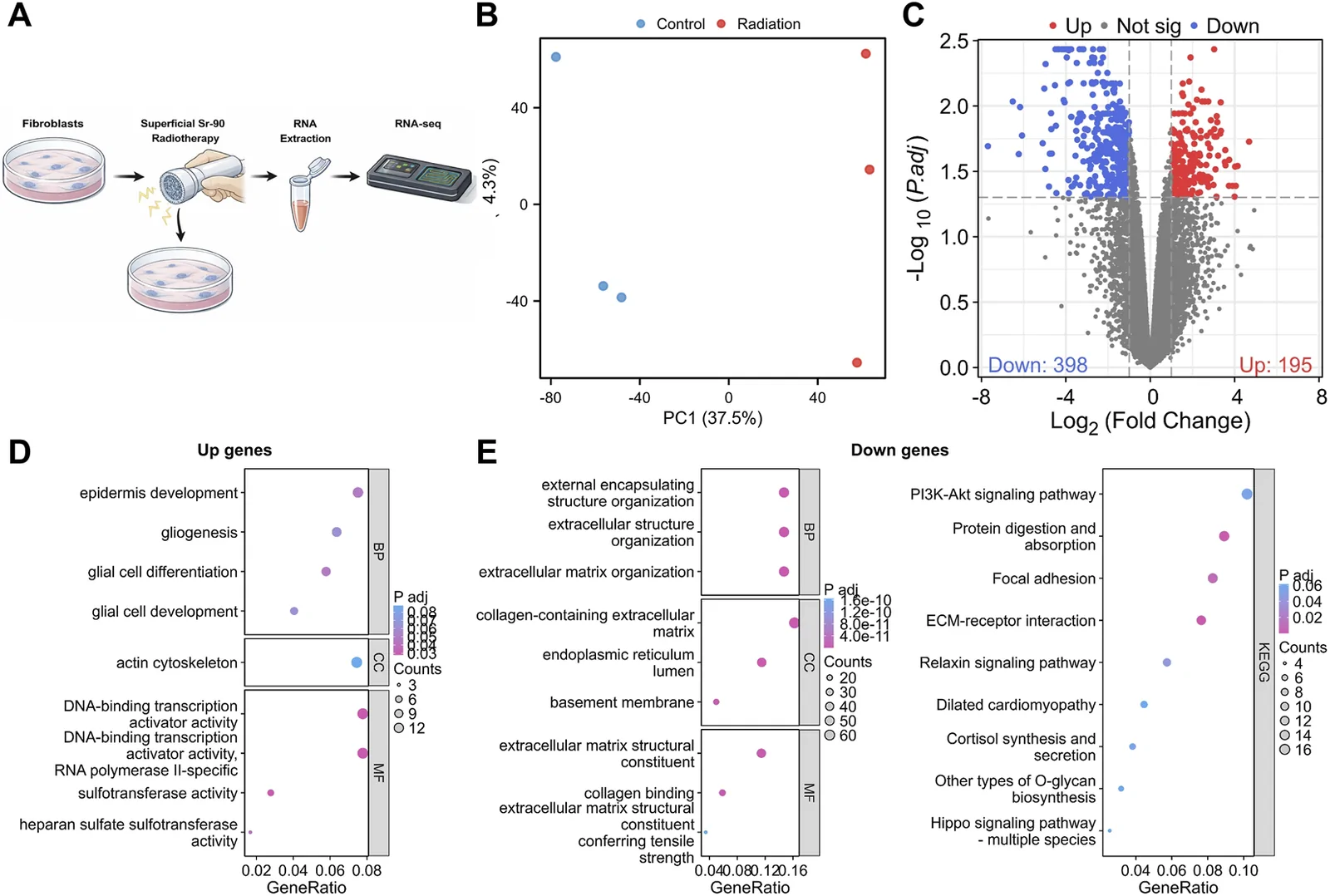
RNA-seq analysis of transcriptomic changes associated with Sr-90 superficial radiotherapy in scar fibroblasts. Note **(A)** Schematic overview of the RNA-seq workflow comparing scar fibroblasts from the Sr-90 superficial radiotherapy group and the control group **(B)** PCA of RNA-seq data showing differences in gene expression distribution between control and irradiated samples **(C)** Volcano plot of differential expression analysis showing the distribution of upregulated and downregulated genes in the radiotherapy group relative to the control group **(D)** GO enrichment analysis of upregulated DEGs after radiotherapy across the BP, CC, and MF categories **(E)** GO and KEGG enrichment analyses of downregulated DEGs after radiotherapy, highlighting their distribution across ECM-related BP and signaling pathways. N = 3 per group.

To systematically define the transcriptional regulatory landscape associated with Sr-90 superficial radiotherapy in scar fibroblasts, RNA sequencing (RNA-seq) was performed on control and irradiated samples (Figure 10A). PCA showed a clear distinction between the two groups, suggesting marked transcriptomic reprogramming after radiotherapy (Figure 10B). Differential expression analysis identified 195 upregulated and 398 downregulated DEGs (Figure 10C).

GO enrichment analysis showed that the upregulated genes were primarily associated with BP related to cell differentiation and development, and were significantly enriched in DNA-binding transcription activator activity at the MF level (Figure 10D). In contrast, the downregulated DEGs were strongly enriched in ECM organization, collagen-associated structures, and cell adhesion-related processes. KEGG analysis further indicated significant enrichment in pathways such as ECM–receptor interaction, focal adhesion, and PI3K-Akt signaling (Figure 10E), consistent with reduced expression of transcriptional programs linked to matrix deposition and mechanotransduction. Together, these findings show that the transcriptomic profile after Sr-90 superficial radiotherapy is characterized by enrichment of transcription-regulatory networks and reduced expression of ECM-associated programs.

Overall, Sr-90 superficial radiotherapy was associated with pronounced transcriptomic reprogramming in scar fibroblasts, characterized by increased expression of transcription-related gene networks and decreased expression of ECM- and adhesion-related pathways. This expression pattern suggests that radiotherapy may participate in the biological regulation of scar tissue by reshaping fibroblast transcriptional control and matrix homeostasis.

## Discussion

The present study supports a working model in which Sr-90 superficial radiotherapy attenuates keloid-related fibrosis in association with parallel changes in fibroblast senescence and ECM remodeling. Our findings show that Sr-90 suppresses the proliferation and migration of keloid fibroblasts and is accompanied by activation of the p53/p21-mediated DDR, senescence-associated changes, and concurrent downregulation of LOX/LOXL2 expression and enzymatic activity, which may contribute to reduced collagen crosslinking-related matrix stabilization and aberrant matrix accumulation. These molecular and cellular changes are accompanied by measurable improvements in tissue architecture and mechanical behavior. Previous studies on radiotherapy for keloids have focused primarily on recurrence control, scar volume reduction, or diminished cellular activity, with limited mechanistic insight into how radiotherapy may simultaneously regulate cell fate and matrix remodeling (Ji et al., 2015; Saini and Gurung, 2025). Our data suggest that the effects of Sr-90 extend beyond growth suppression and involve modulation of the pathological cell-matrix interactions that sustain fibrosis.

At the cellular level, Sr-90 superficial radiotherapy was associated with a senescence-like state in keloid fibroblasts, as reflected by increased SA-β-gal positivity, accumulation of γ-H2AX, and reduced proliferative and migratory capacity. Prior radiobiological studies have tended to emphasize cell death or proliferative arrest, whereas the concept of inducing senescence in pathological fibroblasts has received comparatively little attention as a central therapeutic mechanism (Ji et al., 2015; Saini and Gurung, 2025). The Pifithrin-α experiments support the functional involvement of p53 signaling in these senescence-associated changes and the associated reduction in profibrotic fibroblast activity. Transcriptomic profiling further reinforces this interpretation: genes associated with stress responses and transcriptional regulation were upregulated after radiotherapy, whereas networks linked to proliferation, adhesion, and profibrotic programming were repressed. This pattern shows that Sr-90 irradiation is associated with broad transcriptional changes in fibroblasts rather than a limited phenotypic shift.

At the matrix level, another major finding of this study is that Sr-90 markedly suppresses LOX and LOXL2 expression and activity, accompanied by collagen network loosening, reduced fiber compaction, and decreased tissue stiffness. In contrast to earlier studies that focused largely on total collagen abundance (Kim and Kim, 2024), our data further show that Sr-90 irradiation is associated with altered collagen maturation- and stabilization-related indices. The refractory nature of keloids arises not only from excessive collagen production, but also from matrix hardening and structural fixation driven by LOX-dependent over-crosslinking. Our results show two concurrent matrix-associated changes after Sr-90 irradiation: reduced collagen deposition and attenuation of collagen crosslinking-related matrix stabilization, accompanied by reduced tissue stiffness and fibroblast activation. The transcriptomic downregulation of ECM organization, ECM-receptor interaction, focal adhesion, and PI3K-Akt signaling provides system-level support for this interpretation of matrix remodeling. Importantly, determining which specific properties are targeted by Sr-90 highlights its critical role in modifying ECM microarchitectonics. The pathological keloid ECM differs fundamentally from healthy tissue in both its chemical and physical properties (Kim et al., 2021; Lloyd and He, 2024). Chemically, it is characterized by an abnormally high density of covalent crosslinks, resulting in a low soluble-to-insoluble collagen ratio. Physically, this translates into extreme tissue stiffness, thick collagen bundles, and dense topological compaction (He et al., 2024). Our findings indicate that Sr-90 may modulate these aberrant matrix properties, at least in part, in association with reduced LOX/LOXL2 expression and activity. This interpretation is supported by reduced LOX/LOXL2 expression and activity, altered soluble-to-insoluble collagen fractions, loosened collagen fiber organization, and reduced tissue stiffness. However, direct biochemical inhibition of LOX enzymatic function and direct blockade of specific collagen crosslinks were not demonstrated in the present study and require further validation.

An important feature of this study is the mechanistic framework linking cellular regulation with matrix remodeling. Earlier work has often considered fibroblast hyperactivation and excessive matrix deposition as parallel but separate phenomena (Ji et al., 2015; Poe et al., 2023). Here, Sr-90 was associated with both dimensions of the fibrotic process: senescence-associated changes and reduced pathological fibroblast behavior accompanied by enhanced p53/p21 signaling, as well as reduced collagen crosslinking-related indices and tissue stiffness accompanied by lower LOX/LOXL2 expression and activity. Together, these effects shift scar tissue from a pathologically tense and highly fibrotic state toward one that is comparatively permissive to remodeling. Importantly, the molecular and transcriptomic findings were corroborated by multimodal imaging, ultrastructural analysis, and biomechanical measurements, showing that these parallel molecular and cellular changes were reflected in tangible tissue-level improvements rather than being confined to isolated molecular markers.

The pathway-blocking and combination-intervention experiments provided additional support for this working model. Pifithrin-α attenuated the senescence-associated and antifibrotic changes observed after Sr-90 irradiation, supporting the functional involvement of p53 signaling. By contrast, co-treatment with BAPN further shifted collagen crosslinking-related indices and enhanced tissue remodeling, showing a greater combined effect of LOX inhibition and Sr-90 irradiation. These findings support a combination strategy that targets both aberrantly activated fibroblasts and pathologically stabilized matrix. They also provide an experimental basis for future studies of combination therapy for scar control.

Beyond the fibroblast-ECM axis, the complexity of the immunological response within the keloid microenvironment must not be overlooked. Because the current evaluation focused primarily on fibroblast behavior and matrix mechanics, it remains unclear whether Sr-90-induced ECM remodeling also influences immune-cell distribution, activation, or fibroblast–immune-cell crosstalk within the keloid microenvironment. Keloid pathogenesis is deeply rooted in chronic inflammation, driven by major immune cell subpopulations such as macrophages, mast cells, and neutrophils, as well as their intricate intercellular interactions (Shestakova et al., 2022; Mihlan et al., 2024). Because our current evaluation primarily focused on fibroblast behavior and matrix mechanics, the dynamic interplay between Sr-90 irradiation, local immune cell reprogramming, and immune-mediated ECM regulation remains largely unexplored in this study. Future studies should therefore evaluate immune-cell composition and fibroblast-immune-cell interactions to further clarify the *in vivo* effects of Sr-90.

In summary, Sr-90 superficial radiotherapy remodeled fibroblast behavior, collagen architecture, and tissue biomechanics, accompanied by concurrent senescence-associated changes and attenuation of collagen crosslinking-related matrix stabilization. These findings extend current understanding of Sr-90 from growth suppression to broader regulation of the fibrotic microenvironment. They also provide preliminary mechanistic information for future evaluation of Sr-90 dosing strategies, efficacy assessment, and combination approaches with matrix-targeted agents. Several limitations should nevertheless be acknowledged. *In vitro* systems and animal models cannot fully recapitulate the heterogeneity of clinical keloids. Specifically, the cellular subpopulations that are most sensitive to low-dose irradiation *in vivo* have not yet been definitively identified, although our preliminary findings focus on fibroblasts. Second, although senescence-associated growth arrest may limit fibroblast expansion, radiation-associated senescence and the accompanying SASP-like response can act as a double-edged sword. As recently reported, senescent cells (such as senescent mesenchymal stem cells) undergoing SASP transformation can act as drivers of chronic inflammation and inflammaging, potentially contributing to adverse pathological outcomes (Lyamina et al., 2023). The long-term consequences of accumulating senescent cells and sustained secretion of SASP-associated factors in irradiated tissues therefore require careful investigation. In addition, only a single 2 Gy Sr-90 exposure was used in both the *in vitro* and *in vivo* experiments. Although this design allowed consistent mechanistic evaluation across models, it did not define a dose-response relationship, an optimal biological dose, or a clinically translatable fractionation schedule. Future studies should include graded doses, different fractionation regimens, and longer follow-up periods to determine the dose window that balances antifibrotic efficacy and safety. Finally, additional regulatory networks suggested by the transcriptomic analysis require further validation. Future studies should therefore evaluate the translational value of the identified imaging and biomechanical indices in clinical cohorts, clarify the temporal relationship between cellular senescence and matrix softening, and explore clinically feasible strategies for LOX inhibition or combination radiotherapy, with the ultimate goal of advancing keloid management toward mechanism-driven precision intervention.

## Data Availability

The RNA sequencing data generated in this study have been deposited in the NCBI Sequence Read Archive (SRA) under BioProject accession number PRJNA1507504. The corresponding SRA accession numbers are SRR40013392, SRR40013393, SRR40013394, SRR40013395, SRR40013396, and SRR40013397. The data are publicly available through the NCBI BioProject database.

## Glossary

AFM: Atomic Force Microscopy
ANOVA: Analysis of Variance
α-SMA: Alpha-Smooth Muscle Actin
BAPN: β-Aminopropionitrile
BCA: Bicinchoninic Acid
BP: Biological Process
BSA: Bovine Serum Albumin
CC: Cellular Component
CCK-8: Cell Counting Kit-8
*CDKN1A*: Cyclin-Dependent Kinase Inhibitor 1 A
*COL1A1*: Collagen Type I Alpha 1 Chain
*COL3A1*: Collagen Type III Alpha 1 Chain
DAB: 3,3′-Diaminobenzidine
DAPI: 4′,6-Diamidino-2-Phenylindole
DDR: DNA Damage Response
DEGs: Differentially Expressed Genes
DMEM: Dulbecco’s Modified Eagle Medium
ECM: Extracellular Matrix
ECL: Enhanced Chemiluminescence
EDTA: Ethylenediaminetetraacetic Acid
EdU: 5-Ethynyl-2′-Deoxyuridine
ELISA: Enzyme-Linked Immunosorbent Assay
F-actin: Filamentous Actin
FBS: Fetal Bovine Serum
FDR: False Discovery Rate
GO: Gene Ontology
H&E: Hematoxylin and Eosin
HRP: Horseradish Peroxidase
IF: Immunofluorescence
IHC: Immunohistochemistry
IL-6: Interleukin-6
IL-8: Interleukin-8
KEGG: Kyoto Encyclopedia of Genes and Genome
MF: Molecular Function
OCT: Optical Coherence Tomography
PBS: Phosphate-Buffered Saline
PBS-T: Phosphate-Buffered Saline with Tween-20
PCA: Principal Component Analysis
PVDF: Polyvinylidene Difluoride
QI: Quantitative Imaging
RIN: RNA Integrity Number
RIPA: Radioimmunoprecipitation Assay
RNA-seq: RNA Sequencing
ROI: Region of Interest
RT-qPCR: Reverse Transcription-Quantitative Polymerase Chain Reaction
SA-β-gal: Senescence-Associated β-Galactosidase
SASP: Senescence-Associated Secretory Phenotype
SD: Standard Deviation
SDS-PAGE: Sodium Dodecyl Sulfate-Polyacrylamide Gel Electrophoresis
SEM: Scanning Electron Microscopy
Sr-90: Strontium-90
TMB: 3,3′,5,5′-Tetramethylbenzidine
*TP53*: Tumor Protein p53
WB: Western blot
