# Supplementary material for: Mechanistic insights into Strontium-90 superficial radiotherapy in keloids: senescence-associated changes and ECM remodeling across cell-matrix scales
Source: Front Pharmacol. 2026 Aug 19;17:1832367. doi: 10.3389/fphar.2026.1832367 (PMC13535961; doi:10.3389/fphar.2026.1832367)
Supplement: Supplementary file 1 [file Supplementaryfile1.docx]

Figure 1D-1

Figure 1D-2

Figure 1D-3

Figure 2F-1

Figure 2F-2

Figure 3B-1

Figure 3B-2

Figure 3B-3

Figure 4B-1

Figure 4B-2

Figure 4B-3

Figure 5C-1

Figure 5C-2

Figure 5C-3

Figure 5C-4

Figure 6C-1

Figure 6C-2

Figure 6C-3Figure
